# Where are we in understanding the natural history of polycystic ovary syndrome? A systematic review of longitudinal cohort studies

**DOI:** 10.1093/humrep/deac077

**Published:** 2022-05-10

**Authors:** Sylvia Kiconco, Chau Thien Tay, Kate Louise Rassie, Ricardo Azziz, Helena J Teede, Anju E Joham

**Affiliations:** 1 Monash Centre for Health Research and Implementation, School of Public Health and Preventive Medicine, Monash University, Clayton, Victoria, Australia; 2 Departments of Endocrinology and Diabetes, Monash Health, Clayton, Victoria, Australia; 3 Department of Obstetrics and Gynaecology, School of Medicine, University of Alabama at Birmingham, Birmingham, Alabama, USA; 4 Department of Medicine, School of Medicine, University of Alabama at Birmingham, Birmingham, Alabama, USA; 5 Department of Healthcare Organization & Policy, School of Public Health, University of Alabama at Birmingham, Birmingham, Alabama, USA; 6 Department of Health Policy, Management, and Behaviour, School of Public Health, University at Albany, SUNY, Rensselaer, New York, USA

**Keywords:** polycystic ovary syndrome, PCOS, reproductive, psychological, follow-up, natural history, review

## Abstract

**STUDY QUESTION:**

What is the natural history of reproductive, psychological and oncological features in women with polycystic ovary syndrome (PCOS) in comparison to those without PCOS across the life course?

**SUMMARY ANSWER:**

Existing longitudinal data on changes in reproductive, psychological and oncological features in PCOS are inadequate and conflicting, but the limited evidence suggests that total testosterone (T) and dehydroepiandrosterone sulphate (DHEAS) levels decline more significantly in women with PCOS than in those without PCOS, and the risk of gestational diabetes is higher in pregnant women with PCOS compared to their counterparts without PCOS.

**WHAT IS KNOWN ALREADY:**

The progression of reproductive, psychological and oncological features in PCOS remains unclear, which limits prevention and early diagnosis strategies across the lifespan. Understanding the natural history of PCOS is one of the overarching priorities in PCOS research.

**STUDY DESIGN, SIZE, DURATION:**

This is a systematic review of longitudinal cohort studies with a narrative presentation of findings. Databases MEDLINE, EMBASE, Ovid PsycInfo, CINAHL PLUS and EBM reviews were searched between 15 January 2020 and 11 February 2021 with no language restrictions. Only studies published from the year 1990 to February 2021 were included.

**PARTICIPANTS/MATERIALS, SETTING, METHODS:**

In line with current guidelines for the assessment and management of PCOS, we included studies where participants were females with PCOS diagnosed according to the 2003 Rotterdam or the 1990 National Institutes of Health (NIH) consensus criteria.

**MAIN RESULTS AND THE ROLE OF CHANCE:**

A total of 21 longitudinal studies including 62 123 participants over four continents reported reproductive, psychological and/or oncological outcomes. Participants were females aged between 15 and 49 years at baseline, with follow-up periods ranging from 4 weeks to 32 years. Consistent evidence based on limited studies suggests that total T and DHEAS levels decline to a greater degree in women with PCOS compared to those without PCOS, and the risk gestational diabetes is higher in women with PCOS than in those without PCOS. Evidence reporting changes over time in the majority of the remaining outcomes was unclear due to conflicting and/or insufficient information.

**LIMITATIONS, REASONS FOR CAUTION:**

There was extreme heterogeneity between studies in terms of study setting, population characteristics, follow-up period, effect measures used and laboratory testing approaches.

**WIDER IMPLICATIONS OF THE FINDINGS:**

Understanding the natural history of PCOS and changes in diagnostic, reproductive, psychological and oncological features of PCOS across the lifespan is still a challenge and the existing literature is both limited and conflicting. It is important that future long-term prospective longitudinal studies are conducted in unselected and well-characterized populations.

**STUDY FUNDING/COMPETING INTEREST(S):**

This specific study was not funded. S.K. is supported by scholarships from the Research Training Program of the Commonwealth of Australia and Monash University; H.J.T. is supported by an Australian National Health and Medical Research Council fellowship; and A.E.J. is supported by the Australian National Health and Medical Research Council's Centre for Research Excellence in Women’s Health in Reproductive Life. R.A. was employed by the American Society for Reproductive Medicine and is a consultant to Spruce Biosciences and Fortress Biotech. The other authors have no conflicts of interest to declare.

**REGISTRATION NUMBER:**

Prospero registration number: CRD42020165546.

## Introduction

Polycystic ovary syndrome (PCOS) is an endocrine-metabolic disorder diagnosed in adults based on the presence of at least two of three clinical features, including polycystic ovary morphology, oligo/amenorrhea and hyperandrogenism (clinical and/or biochemical). PCOS is a major public health issue that affects 5–15% women of reproductive age globally (Azziz *et al.*, 2006; Diamanti-Kandarakis *et al.*, 2006; March *et al.*, 2010; Bozdag *et al.*, 2016) but is complicated by diagnostic challenges including a lack of clear definitions for individual PCOS features. These contribute to misdiagnosis, delayed diagnosis and patient dissatisfaction (Dokras *et al.*, 2017; Gibson-Helm *et al.*, 2017), and up to 70% of women with the condition remain undiagnosed (March *et al.*, 2010). The lack of information on changes in biochemical and clinical hyperandrogenism, cycle regularity and ovarian morphology, including the PCOS phenotype over the lifespan, also complicates diagnosis and warrants further investigation.

Women with PCOS are at increased risk of adverse reproductive, metabolic and psychological outcomes. Common reproductive features of the condition include biochemical hyperandrogenism, ovulatory and menstrual dysfunction, hirsutism, subfertility, endometrial hyperplasia and obstetrical complications (Teede *et al.*, 2018a,b). Women with PCOS are also at a higher risk of infertility or reduced fertility than those without PCOS, which may be driven by changes in oocyte, endometrial and embryo function (Palomba, 2021). Metabolic features include increased risks for insulin resistance, dyslipidemia, impaired glucose tolerance, metabolic syndrome, gestational diabetes, type 2 diabetes and cardiovascular disease (Legro *et al.*, 1999; Apridonidze *et al.*, 2005). Psychological features include anxiety, depression, low self-esteem and poor body image (Moran *et al.*, 2010; Teede *et al.*, 2010; Deeks *et al.*, 2011). Increased endometrial cancer risk has also been associated with PCOS (Charalampakis *et al.*, 2016). These diverse PCOS features lead to a diminished quality of life in affected women (Dokras *et al.*, 2018; Teede *et al.*, 2018a,b). Overall, PCOS is associated with a substantial economic burden, conservatively estimated to exceed an annual total cost of $8 billion USD in the USA alone (2020 USD), including healthcare costs related to diagnosis, reproductive, metabolic, vascular and pregnancy-related morbidities (Riestenberg *et al.*, 2022).

The recent international evidence-based guideline for the Diagnosis and Management of PCOS (Teede *et al.*, 2018b) highlighted our limited understanding of the natural history of reproductive, psychological and oncological outcomes in PCOS and identified major gaps that currently limit the development of effective prevention strategies across the lifespan. Furthermore, understanding the natural history of PCOS emerged from the guideline process as one of the overarching priorities in PCOS research. Therefore, we now aim to explore the natural history of PCOS with a focus on reproductive and psychologic features as well as cancer risk, by conducting a systematic review of longitudinal cohort studies.

## Materials and methods

### Protocol

This review was conducted following the Preferred Reporting Items for Systematic Reviews and Meta-Analysis (PRISMA) guidelines (Moher *et al.*, 2010; Page *et al.*, 2021) and was registered (CRD42020165546) in the international prospective register of systematic reviews (PROSPERO).

### Literature search

#### Search strategy

A comprehensive systematic search based on the selection criteria combining MeSH terms and text words was developed using the OVID platform and translated to the CINAHL database as appropriate (Supplementary Data File S1). The search terms used were based on the harmonized core outcomes set for PCOS (Al Wattar *et al.*, 2020) and the search was limited to human studies published from the year 1990 to 11 February 2021. The 1990 limit reflects the establishment of the first modern definition for PCOS, the National Institutes of Health (NIH) 1990 criteria (Zawadski and Dunaif, 1992). Studies were included regardless of the publication language.

#### Databases

Various electronic databases were first searched on 15 January 2020 and the search was updated on 11 February 2021. Specifically, these databases included Ovid MEDLINE(R) 1946 to 07 January 2020, EMBASE Classic+Embase (1947 to 07 January 2020), PsycINFO (1806 to December Week 5 2019) and CINAHL PLUS via the EBSCO host Interface, as well as all EBM Reviews.

### Inclusion and exclusion criteria

The Participant, Intervention, Comparison, Outcome and Study type (PICOS) framework (Supplementary Table SI) was used in selection of articles included in this review. Participants were females of any age group and any weight with a PCOS diagnosis according to the 2003 Rotterdam or the 1990 NIH consensus criteria, to align with current international evidence-based guidelines for the assessment and management of PCOS (Teede *et al.*, 2018b). Females without PCOS (any age group and weight) were considered as the comparison group. Both retrospective and prospective longitudinal cohort studies were included. Studies were excluded if the participants’ PCOS diagnostic criteria were not NIH or Rotterdam 2003, or were unclear such as use of ICD codes alone.

In terms of intervention/exposure, this review included studies that followed women with a PCOS diagnosis and reported longitudinal findings that demonstrated changes or predicted risk for specific PCOS features or outcomes over time (without treatment, to reflect the natural history of the condition). Follow-up studies with or without a comparison group of women without PCOS were also included. Main reproductive, metabolic, psychological and oncological-related outcomes were categorized in accordance with the PCOS core outcomes set (Al Wattar *et al.*, 2020).

The reproductive category included measures of clinical and biochemical hyperandrogenism, including hirsutism, as measured by the modified Ferriman-Gallwey (mFG) score, testosterone (T), sex hormone-binding globulin (SHBG), free androgen index (FAI), androstenedione (A_4_) and dehydroepiandrosterone sulphate (DHEAS). We also assessed the reproductive hormone profile, including LH, FSH, LH:FSH ratio and anti-Müllerian hormone (AMH). This was in addition to ovulatory function, such as menstrual regularity and chronic anovulation, as well as pregnancy viability and outcomes, including gestational weight gain, gestational diabetes, hypertensive disease in pregnancy, miscarriage, stillbirth, live birth, preterm birth, birth weight, major congenital abnormalities and neonatal mortality. Also assessed were psychological outcomes, including depression, anxiety and eating disorders, and oncological-related outcomes, including atypical endometrial hyperplasia and endometrial cancer.

### Study selection and risk of bias assessment

Screening of articles on abstracts and full text was carried out by two independent reviewers (S.K. and C.T.T. or K.L.R.) to identify eligible studies. Discrepancies were resolved through consensus or by a third reviewer (K.L.R. or C.T.T.). Methodological quality and risk of bias of included studies were assessed by two independent reviewers (S.K. and K.L.R.) using criteria established according to the Monash Centre for Health Research and Implementation (MCHRI) Evidence Synthesis Program critical appraisal tool (Monash Centre for Health Research and Implementation, 2003). The MCHRI critical appraisal tool is based on the Newcastle‐Ottawa Scale (NOS) for non‐randomized studies (Wells *et al.*, 2014). Studies were assessed on individual criteria related to external validity (methodology, inclusion/exclusion criteria and appropriateness of measured outcomes) and internal validity (attrition, detection, selection and reporting bias, confounding, statistical analyses and study power). Studies that fulfilled all, most or few criteria were deemed to have low, moderate and high levels of bias, respectively. Risk of bias assessment was conducted using Covidence software (Babineau, 2014) and disagreements were resolved through discussion to reach a consensus.

### Data extraction and synthesis

Data for each outcome were extracted manually using a researcher pre-designed data extraction form in Microsoft Excel. Data were extracted by two reviewers (S.K. and K.L.R.). Information was collected on general details (authors, reference/source, country, year of publication, setting), participants (age, ethnicity, selection criteria, comparison/subgroups, number of participants, duration of follow-up, PCOS criteria), results (point estimates and measures of variability/effect) and any other key PCOS outcome results related to natural history. A narrative description of results is presented according to each outcome category.

## Results

### Search results

**Figure 1.**
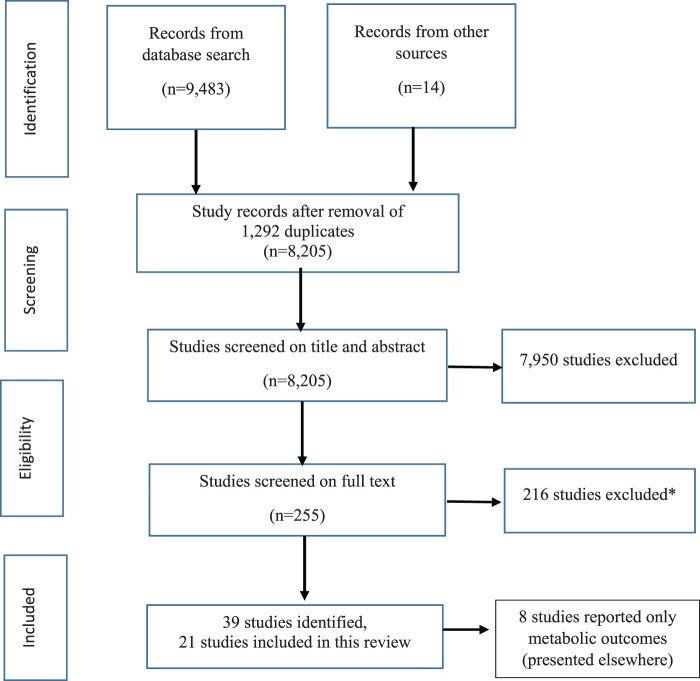
**Flow chart of study selection.** *Reasons for exclusion are in Supplementary Table SII.

A total of 9497 studies were identified from the search (Fig. 1). After exclusion of duplicates (about 10% of studies identified), 8205 and 255 studies were assessed on abstract and full text, respectively. There were 216 studies excluded at full-text review stage due to various reasons, such as unclear criteria or self-reported PCOS status, ineligible outcomes or cross-sectional design, as shown in Supplementary Table SII. Therefore, 39 studies met our inclusion criteria and 21 of them reported reproductive, psychologic and/or oncological-related outcomes. The remaining eight studies reported metabolic outcomes only and have been presented in a separate manuscript (Kiconco *et al.*, 2022).

### Characteristics of included studies

Characteristics of the included studies are outlined in Table I. Six of the studies (Kerchner *et al.*, 2009; Huddleston *et al.*, 2017; Ahmad *et al.*, 2018; Greenwood *et al.*, 2019a,b; Jarrett *et al.*, 2020) were conducted in the USA, four studies were conducted in Italy (Palomba *et al.*, 2007; Carmina *et al.*, 2012a,b; Palomba *et al.*, 2014), three studies (Schmidt *et al.*, 2011a,b; Forslund *et al.*, 2021) were conducted in Sweden, and two were from Denmark (Altinok *et al.*, 2014; Udesen *et al.*, 2019). The remaining six studies were conducted in Hong Kong (Ng *et al.*, 2019), the Netherlands (Brown *et al.*, 2011), Mexico (Reyes-Munoz *et al.*, 2012), Venezuela (Jakubowicz *et al.*, 2002) and Taiwan (Cheng-Che *et al.*, 2015; Harnod *et al.*, 2020).

**Table I:** Characteristics of included studies.

Study	Country	Design	Setting	PCOS group	Non-PCOS group	PCOS criteria	Follow-up duration	Outcomes measured	Risk of bias
Ahmad *et al.* (2018)	USA	Prospective cohort	Academic practice (PCOS clinic)	Age, 30.9 ± 6.46 years n = 31	Age, 36.06 ± 5.36 years n = 267	Rotterdam	PCOS (3.21 ± 1.62), controls 3.90 ± 0.79 years (2007 through 2013)	AMH	Moderate

Altinok *et al.* (2014)	Denmark	Retrospective	Academic practice (out-patient) and population (controls)	Age, 29 years n = 1124	Age, 29 n = 4213	Rotterdam	6.8 (PCOS) and 7.2 (controls) years (1997 to 2012)	Antidepressant prescription	Moderate

Brown *et al.* (2011)	Netherlands	Retrospective	Academic practice (out-patient clinic)	Age, 28.6 years, n = 254	Age, 29.9 years, n = 41	Rotterdam	7 years (1991 through 2009)	LH, FSH, LH/FSH, AMH, total T, DHEAS, FAI, SHBG	High

Carmina *et al.* (2012b)	Italy	Prospective	Academic practice	Age, 37 ± 1 years, n = 54	Age, 37 ± 1 years, n = 20	Rotterdam	5 years (period not specified)	LH, LH/FSH ratio, total T, DHEAS, AMH	Moderate

Carmina *et al.* (2012a)	Italy	Prospective	Academic practice (Endocrine Unit)	Age, 21.9 ± 2.1 years, n = 193	Age, 21.9 ± 2.1 years, n = 35 (controls were not followed up)	Rotterdam	20 years (baseline 1985/1990)	LH: FSH ratio, total T, DHEAS	High

Cheng-Che *et al.* (2015)	Taiwan	Retrospective	National health insurance registry	Age, 27 years, n = 3566	Age, 7 years, n = 14 264	NIH	7.15 years (2000/2004 to 2009)	Breast cancer, and uterine cancer	Low

Forslund *et al.* (2021)	Sweden	Prospective cohort	Academic practice	Age 49.4 ± 5.0 years, n = 21	Age 49.7 ± 5.6 years, n = 55	Rotterdam	32 years (1987 to 2019)	FSH, LH, DHEAS, SHBG, T, A_4_, FAI	Moderate

Greenwood *et al.* (2019a)	USA	Prospective	Academic practice (PCOS clinic)	Age, 29 years n = 163	No controls	Rotterdam	5.5 years (2006–2017-consecutive)	BDI-FS score	Moderate

Greenwood *et al.* (2019b)	USA	Prospective	General population	Age 23 to 35 years, n = 83	Age 23 to 35 years, n = 1044	NIH	30 years at 5 year intervals (recruited 1985/1986)	CES-Depression symptom scores	Low

Harnod *et al.* (2020)	Taiwan	Retrospective cohort	Health insurance database	Age 27.74 ± 6.8 years, n = 7026	Age 27.74 ± 6.8 years, n = 28 104	NIH or Rotterdam	16 years (1996 to 2013)	Incident anxiety	Moderate

Huddleston *et al.* (2017)	USA	Prospective	Academic practice (PCOS clinic)	Age, 30.4 ± 5.6 years, n = 38	Age, 35.7 ± 5.5 years, n = 296	Rotterdam	3-4 years (2004–2014 cohort)	Total T	Low

Jarrett *et al.* (2020)	USA	Prospective cohort	General population	Age 26 ± 4 years, n = 26	Age 30 ± 6 years, n = 12	NIH	4 to 6 weeks (at every other day interval)	LH, FSH	High

Jakubowicz *et al.* (2002)	Venezuela	Retrospective nested in RCT	Hospital (Endocrinology Clinic)	Age 30.0 ± 3.2, years, n = 31	No controls	NIH	4.5 (1996–2000)	Free T, early pregnancy loss, pre-term births	High

Kerchner *et al.* (2009)	USA	Prospective	Academic practice (PCOS clinic)	Age 32 ± 6.3 years, n = 60	No controls	Rotterdam	22 ± 3.7 months (baseline 1997/1999)	Depression	High

Ng *et al.* (2019)	Hong Kong	Prospective cohort	General hospital and community (controls)	Age 30.6 ± 6.5 years, n = 199	Age 42.6 ± 7.0 years, n = 242	Rotterdam	10.6 ± 1.3 years (2003/2007 to 2016/2017)	FSH, LH, total T, FAI, AMH	Low

Palomba *et al.* (2014)	Italy	Prospective	Academic practice (Obstetrics and Gynecology department)	Age 27.8 ± 3.6 years, n = 150, Gestational age (5.4 weeks)	Age 27.4 ± 4.0 years, n = 150 Gestational age (5.4 weeks)	Rotterdam	27 gestational weeks (2003 through 2012)	Total T, A, DHEAS, SHBG, FAI, miscarriage, PIH, PE, GDM, B/W	Low

Palomba *et al.* (2007)	Italy	Prospective nested in RCT	Academic practice	Age 24.8 ± 2.7 years, n = 13	Age 25.6 ± 2.7 years, n = 10	NIH	24 months (recruited 2003/2004 to 2005/2006)	mFG score, total T, A_4_, DHEAS, SHBG, FAI	Moderate

Reyes-Munoz *et al.* (2012)	Mexico	Prospective	Academic practice	Age 29.1 ± 3.9 years, n = 52	Age 29.0 ± 3.8 years, n = 52	Rotterdam	29.4 gestational weeks (2006 January through to December 2007	GDM, miscarriage, preterm birth, pre-eclampsia, stillbirth, congenital malformation, weight gain, newborn weight	High

Schmidt *et al.* (2011a)	Sweden	Prospective	Academic practice	Age 49.4 ± 4.9 years, n = 25	Age 49.7 ± 5.6 years, n = 68	Rotterdam	21 years (1987–2008)	Menopause age, FSH, LH, SHBG, total T, A_4_, DHEAS, FAI	Moderate

Schmidt *et al.* (2011b)	Sweden	Prospective	Academic practice	Age 49.4 ± 4.9 years, n = 25	Age 49.7 ± 5.6 years, n = 68	Rotterdam	21 years (1987–2008)	Breast, and endometrial cancers	Moderate

Udesen *et al.* (2019)	Denmark	Prospective	Fertility clinic	Age 29.1 ± 4.1 years, n = 40	Age 30.0 ± 5.2 years, n = 8	Rotterdam	5.8 ± 0.8 years (recruited 2010/2012)	mFG score, total T, free T, DHEAS, A_4_, SHBG, LH/FSH ratio	High

A_4_, androstenedione; AMH, anti*-*Müllerian hormone; B/W, birth weight; BDI-FS, Beck Depression Inventory Fast Screen; CES-D, Center for Epidemiologic Studies-Depression; DHEAS, dehydroepiandrosterone sulphate; FAI, free androgen index; GDM, gestational diabetes mellitus; HTN, hypertension; mFG, modified Ferriman-Gallwey score; PCOS, polycystic ovary syndrome; PE, pre-eclampsia; PIH, pregnancy-induced hypertension; SHBG, sex hormone-binding globulin; T, testosterone.

The baseline age of participants ranged from 21 to 37 years for 18 of the studies, while the baseline age in three studies (Schmidt *et al.*, 2011a,b; Forslund *et al.*, 2021) was 49 years. The mean follow-up duration ranged from 4 weeks to 32 years (study-combined average of 4 to 16 years per parameter) for non-pregnancy-related outcomes (Supplementary Fig. S1), and from 24 to 27 gestational weeks for pregnancy-associated outcomes. Only one study obtained participants from a nationally linked database (Cheng-Che *et al.*, 2015), while the rest of the studies were practice-based (general hospital or academic). Four studies (Jakubowicz *et al.*, 2002; Kerchner *et al.*, 2009; Carmina *et al.*, 2012a; Greenwood *et al.*, 2019a) were uncontrolled or the control participants were not followed up. The assigned overall risk of bias was low for 5 of the 21 (23.8%) studies (Palomba *et al.*, 2014; Cheng-Che *et al.*, 2015; Ng *et al.*, 2019; Greenwood *et al.*, 2019b) and the rest demonstrated moderate (n = 9) or high (n = 7) risk (Table I).

Thirteen studies (Jakubowicz *et al.*, 2002; Palomba *et al.*, 2007; Brown *et al.*, 2011; Schmidt *et al.*, 2011a; Carmina *et al.*, 2012a,b; Palomba *et al.*, 2014; Huddleston *et al.*, 2017; Ahmad *et al.*, 2018; Ng *et al.*, 2019; Udesen *et al.*, 2019; Jarrett *et al.*, 2020; Forslund *et al.*, 2021) reported data on reproductive outcomes (hormonal profiles, clinical and biochemical hyperandrogenism and menstrual cycle regularity). Three studies (Jakubowicz *et al.*, 2002; Reyes-Munoz *et al.*, 2012; Palomba *et al.*, 2014) reported pregnancy-related outcomes, five reported on psychological outcomes (Kerchner *et al.*, 2009; Altinok *et al.*, 2014; Greenwood *et al.*, 2019a,b; Harnod *et al.*, 2020) and two reported oncological-related outcomes (Schmidt *et al.*, 2011b; Cheng-Che *et al.*, 2015). The detailed data on observed changes over time for each of the outcomes are in Tables II–IV.

**Table II:** Changes in reproductive outcomes over time.

Outcomes		Study author, year	Baseline age, sample size		Mean follow-up duration	Effect measures	Observed estimates		
			PCOS group	Non-PCOS group			Within PCOS group comparison	Within non-PCOS group comparison	PCOS group versus non-PCOS group
Clinical hyperandrogenism	Hirsutism (mFG score)	Palomba *et al.* (2007)	Age 24.8 years, n = 13	Age 25.6 years, n = 10	24 months	Mean comparison with baseline (24,18 months vs 6 months)	10.8 ± 1.8, 10.8 ± 2.2 vs 10.7 ± 1.8, *P* > 0.05	4.5 ± 1.4, 4.6 ± 1.4 vs 4.4 ± 1.6, *P* > 0.05	*P* < 0.05 at 18th and 24th visits (changes between groups not compared)
		
		Udesen *et al.* (2019)	Age 29.1 years, n = 40	Age 30.0 years, n = 8	5.8 ± 0.8 years	Mean comparison with baseline	5.0 (3.0–10.0) vs 6.0 (3.0–9.0), NS	2.5 (1.5–4.5) vs 1.5 (0–5.5), NS	–
	
	Acne	–	–	–	–	–	–	–	–
	
	Hair loss	–	–	–	–	–	–	–	–

Biochemical hyperandrogenism	Total T	Carmina *et al.* (2012a), Carmina *et al.* (2013)	Age 21.8 years, n = 193	Age 21.8 years, n = 35 (not followed up)	20 years	Mean comparisons (5th to 20th years) with baseline (ng/dl)	59 ± 28, 65 ± 25, 68 ± 22 vs 75 ± 26 (*P* < 0.05)	–	–
		
		Huddleston *et al.* (2017)	Age 30.4 years, n = 38	Age 35.7 years, n = 296	3–4 years	Change per year (nmol/l)	BMI >30: [−0.09 (95% CI −0.16 to −0.02)], *P* < 0.05 BMI ≤ 30: [−0.04 (95 % CI −0.11 to 0.03)], NS	Not reported	–
		
		Schmidt *et al.* (2011a)	Age 49.4 years, n = 25	Age 49.7 years, n = 68	21 years	Mean change from baseline (nmol/l)	−0.82 ± 0.88, *P* = 0.001	−0.36 ± 0.74, *P* = 0.001	*P* = 0.016
		
		Udesen *et al.* (2019)	Age 29.1 years, n = 40	Age 30.0 years, n = 8	5.8 years	Median comparison with baseline nmol/l	1.4 (1.0 to 2.0) vs 1.9 (1.4 to 2.5), *P* < 0.001	0.7 (0.4 to 0.9) vs 0.9 (0.7 to 1.6), *P* = 0.039	–
		
		Ng *et al.* (2019)	Age 30.6 years, n = 199	Age 42.6 years, n = 242	10.6 years	Change from baseline (nmol/l)	−0.5 ± 0.7 *P* < 0.001	–	–
		
		Forslund *et al.* (2021)	Age 49.4 years, n = 21	Age 49.7 6 years, n = 55	32 years	Change from baseline (nmol/l)	–1.2 ± 1.1, *P* < 0.01	–0.8 ± 0.7, *P* < 0.01	*P* = 0.48
		
		Palomba *et al.* (2007)	Age 24.8 years, n = 13	Age 25.6 years, n = 10	24 months	Mean comparison with baseline (24.18 vs 6 months)	1.5 ± 0.5, 1.6 ± 0.4 vs 1.7 ± 0.3 (ng/ml), *P* > 0.05	0.6 ± 0.2, 0.6 ± 0.2 vs 0.6 ± 0.2 (ng/ml), *P* > 0.05	*P* < 0.05 at 18th and 24th visits (changes between groups not compared)
		
		Palomba *et al.* (2014)	Age 27.8 years, n = 150, 5.4 gestational weeks	Age 27.4 years, n = 150 5.4 gestational weeks	27 gestational weeks	Mean comparison with baseline (32nd, 20th weeks vs pre study)	3.6 ± 2.4, 3.8 ± 1.5 vs 3.1 ± 2.3 (ng/dl), *P* < 0.05	1.0 ± 0.2, 1.0 ± 0.2 vs 0.9 ± 0.2 (ng/dl), NS	*P* < 0.01 at all visits (changes between groups not compared)
		
		Carmina *et al.* (2012b)	Age 37 years, n = 54	Age 37 years, n = 20	5 years	Mean comparison with baseline (nmol/l)	58 ± 19 vs 74 ± 22, *P* < 0.01	25 ± 16 vs 28 ± 11, NS	–
		
		Brown *et al.* (2011)	Age 28.6 years, n = 254	Age 29.9 years, n = 41	7 years	Median change per year (ng/ml)	−2.3 (−2.9 to −1.4), *P* < 0.05	<0.0001 (−0.0001 to 0.0001), NS	*P* < 0.001
	Free T	Jakubowicz *et al.* (2002)	Age 30.0 years, n = 31	No controls	4.5 years	Mean comparisons baseline vs 6-week gestation, (ng/dl)	3.3 ± 0.2 vs 3.4 ± 0.3, NS	–	–
		
		Udesen *et al.* (2019)	Age 29.1 years, n = 40	Age 30.0 years, n = 8	5.8 ± 0.8 years	Mean comparison with baseline (nmol/l)	0.032 (0.019 to 0.050) vs 0.023 (0.014 to 0.036), *P* = 0.008	0.019 (0.012 to 0.023) vs 0.012 (0.007 to 0.014), NS	
	
	FAI	Brown *et al.* (2011)	Age 28.6 years, n = 254	Age 29.9 years, n = 41	7 years	Median change per year	<0.0001 (−0.0001 to 0.0001), NS	–	–
		
		Palomba *et al.* (2007)	Age 24.8 years, n = 13	Age 25.6 years, n = 10	24 months	Mean comparison with baseline (24.18 vs 6 months)	22.7 ± 5, 23.5 ± 4.3 vs 22.4 ± 5.7 (%), NS	4.3 ± 1.8, 4.2 ± 2.0 vs 4.5 ± 1.9 (%), NS	*P* < 0.05 at 18th and 24th visits (changes between groups not compared)
		
		Palomba *et al.* (2014)	Age 27.8 years, n = 150, 5.4 weeks gestational	Age 27.4 years, n = 150, 5.4 weeks of gestation	27 gestational weeks	Mean comparison with baseline (32nd, 20th weeks vs pre study)	11.3 ± 3.4, 10.5 ± 3.4 vs 13.0 ± 3.5 (%), *P* < 0.05	3.3 ± 2.5, 3.4 ± 2.1 vs 4.3 ± 2.6 (%), *P* < 0.05	*P* < 0.01 at all visits (changes between groups not compared)
		
		Schmidt *et al.* (2011a)	Age 49.4 years, n = 25	Age 49.7 years, n = 68	21 years	Mean change from baseline	−3.40 ± 4.45, *P* = 0.001	−1.63 ± 2.69, *P* = 0.001	*P* = 0.033
		
		Forslund *et al.* (2021)	Age 49.4 years, n = 21	Age 49.7 years, n = 55	32 years	Change from baseline	–4.5 ± 4.3, *P* < 0.01	–2.4 ± 2.7, *P* < 0.01	*P* = 0.08
		
		Ng *et al.* (2019)	Age 30.6 years, n = 199	Age 42.6 years, n = 242	10.6 years	Change from baseline	−3.0 ± 6.5, *P* < 0.001	–	–
	
	DHEAS	Brown *et al.* (2011)	Age 28.6 years, n = 254	Age 29.9 years, n = 41	7 years	Median change per year (mg/ml)	−0.13 (−0.15 to −0.10), *P* < 0.001	<0.0001 (−0.0001 to 0.0001), NS	*P* < 0.001
		
		Carmina *et al.* (2012a)	Age 21.9 years, n = 193	Not followed up	20 years	Mean comparisons (5th–20th years) with baseline, (µg/ml)	2.00 ± 0.9, 2.1 ± 0.85, 2.2 ± 1.3 vs 2.7 ± 1.2, *P* < 0.01	–	–
		
		Carmina *et al.* (2012b)	Age 37 years, n = 54	Age 37 years, n = 20	5 years	Mean comparisons with baseline (µg/ml)	2.2 ± 1.2 vs 2.4 ± 1, *P* < 0.01	1.7 ± 1 vs 1.8 ± 1, NS	–
		
		Forslund *et al.* (2021)	Age 49.4 years, n = 21	Age 49.7 years, n = 55	32 years	Mean change from baseline, (μmol/l)	–2.9 ± 2.3, *P* < 0.01)	controls –1.4 ± 0.9, *P* < 0.01	*P* = 0.01
		
		Palomba *et al.* (2007)	Age 24.8 years, n = 13	Age 25.6 years, n = 10	24 months	Mean comparison with baseline, (ng/ml)	2579.2 ± 442.2, 2579.2 ± 368.5 vs 2616.1 ± 368.5, *P* > 0.05	1400.1 ± 405.3, 1326.5 ± 331.6 vs 1510.7 ± 368.5, *P* > 0.05	*P* < 0.05 at 18th and 24th visits (changes between groups not compared)
		Palomba *et al.* (2014)	Age 27.8 years, n = 150, 5.4 gestational weeks	Age 27.4 years, n = 150 5.4 gestational weeks	27 gestational weeks	Mean comparison with baseline (32nd, 20th weeks vs pre study), (ng/dl)	2762.1 ± 974.4, 2746.4 ± 977.3 vs 2684.0 ± 981.3, *P* < 0.05	1721.4 ± 713.3 1737.2 ± 722.4 vs 1709.2 ± 733.1, *P* > 0.005	*P* < 0.05 at all visits (changes between groups not compared)
		
		Schmidt *et al.* (2011a)	Age 49.4 years, n = 25	Age 49.7 years, n = 68	21 years	Change from baseline, (µmol/l)	−2.51 ± 1.58, *P* = 0.001	−1.71 ± 1.26, *P* = 0.001	*P* = 0.008
		
		Udesen *et al.* (2019)	Age 29.1 years, n = 40	Age 30.0 years, n = 8	5.8 years	Mean comparison with baseline, (Umol/l)	4857 ± 2190 vs 5676 ± 2764, *P* = 0.017	5181 ± 1962 vs 4437 ± 2550, NS	–
	
	A_4_	Brown *et al.* (2011)	Age 28.6 years, n = 254	Age 29.9 years, n = 41	7 years	Median change per year, (ng/dl)	−17.5 (−21.8, −12.9), *P* < 0.001	–	–
		
		Palomba *et al.* (2014)	Age 27.8 years, n = 150, 5.4 gestational weeks	Age 27.4 years, n = 150 5.4 gestational weeks	27 gestational weeks	mean Comparison with baseline (32nd, 20th weeks vs pre study), (ng/ml)	4.4 ± 3.6, 4.3 ± 3.7 vs 4.2 ± 3.5, *P* > 0.05	1.7 ± 1.3, 1.8 ± 1.0 vs 1.6 ± 1.1, *P* > 0.05	*P* < 0.05 at all visits (changes between groups not compared)
		
		Palomba *et al.* (2007)	Age 24.8 years, n = 13	Age 25.6 years, n = 10	24 months	Mean comparison with baseline (24.18 vs 6 months), (ng/ml)	1.8 ± 0.3, 1.9 ± 0.3 vs 1.9 ± 0.3, *P* > 0.05	0.6 ± 0.1, 0.7 ± 0.02, vs 0.6 ± 0.1, *P* > 0.05	*P* < 0.05 at all visits (changes between groups not compared)
		
		Schmidt *et al.* (2011a)	Age 49.4 years, n = 25	Age 49.7 years, n = 68	21 years	Change from baseline, (nmol/l)	−0.56 ± 3.58, *P* = 0.230	1.82 ± 2.91, *P* = 0.001	*P* = 0.001
		
		Udesen *et al.* (2019)	Age 29.1 years, n = 40	Age 30.0 years, n = 8	5.8 years	Median comparison with baseline, (nmol/l)	5.8 (4.0–8.5) vs 7.1 (4.7–9.0), *P* = 0.048	2.8 (1.8–3.4) vs 4.4 (3.0–5.7), *P* = 0.001	–
		
		Forslund *et al.* (2021)	Age 49.4 years, n = 21	Age 49.7 years, n = 55	32 years	Mean change from baseline, (nmol/l)	–1.5 ± 4.7 *P* = 0.23	0.1 ± 1.7, *P* = 1.00	*P* = 0.17
	
	SHBG	Brown *et al.* (2011)	Age 28.6 years, n = 254	Age 29.9 years, n = 41	7 years	Median change per year, (mg/dl)	−0.03 (−0.05 to −0.001), *P* < 0.01	–	–
		
		Palomba *et al.* (2014)	Age 27.8 years, n = 150, 5.4 gestational weeks	Age 27.4 years, n = 150 5.4 gestational week	27 estational weeks	Mean comparison with baseline (32nd, 20th weeks vs pre study), (nmol/l)	25.0 ± 8.3, 24.7 ± 9.2 vs 18.0 ± 9.5, *P* > 0.05	48.2 ± 14.2, 49.0 ± 13.9 vs 42.7 ± 14.8, *P* > 0.05	*P* < 0.05 at all visits (changes between groups not compared)
		
		Palomba *et al.* (2007)	Age 24.8 years, n = 13	Age 25.6 years, n = 10	24 months	Mean comparison with baseline (24,18 vs 6 months), (nmol/l)	26.3 ± 4.1, 25.9 ± 4.3 vs 26.1 ± 3.5, *P* > 0.05	47.8 ± 7.2, 47.6 ± 6.8 vs 47.5 ± 6.8, *P* > 0.05	*P* < 0.05 at all visits (changes between groups not compared)
		Schmidt *et al.* (2011a)	Age 49.4 years, n = 25	Age 49.7 years, n = 68	21 years	Change from baseline, (nmol/l)	8.9 ± 29.4, *P* = 0.076	12.3 ± 32.5, *P* = 0.001	*P* = 0.290
		
		Udesen *et al.* (2019)	Age 29.1 years, n = 40	Age 30.0 years, n = 8	5.8 years	Median comparison with baseline, (nmol/l)	46.0 (36.5–83.0) vs 59.5 (40.0–83.0), *P* = 0.011	62.5 (45.0–73.5) vs 49.0 (30.0–72.5), NS	–
		
		Forslund *et al.* (2021)	Age 49.4 years, n = 21	Age 49.7 years, n = 55	32 years	Mean change from baseline, (nmol/l)	35 ± 20, *P* < 0.01	33 ± 56, *P* = 0.01	*P* = 0.75

Reproductive hormonal profile	AMH	Ahmad *et al.* (2018)	Age 30.9 years, n = 31	Age 36 years, n = 267	PCOS: 21 controls: 3.9 years	Rate of change/year and % change from baseline, (ng/m/l/year)	Rate/year, 2.96 ± 5.6 % change, −35.8% (−47.5% to −24.0%)	Rate/year 0.29 ± 0.56 % change, 8.0% (−12.0% to −3.9%)	Difference of −2.28 [−3.18 to −1.38]; *P* *<* 0.01 % change −35.8% vs. −8.0%; *P* *<* 0.01
		
		Brown *et al.* (2011)	Age 28.6 years, n = 254	Age 29.9 years, n = 41	7 years	Median change per year, (ng/ml)	<0.0001 (95% CI −0.0001, 0.0001), NS	<0.0001 (95% CI −0.0001, 0.0001), NS	NS
		
		Carmina *et al.* (2012b)	Age 37 years, n = 54	Age 37 years, n = 20	5 years	Mean comparisons with baseline, (ng/ml)	3.9 ± 1.2 vs 6.7 ± 2.1, *P* < 0.01	1 ± 0.7 vs 1.7 ± 0.7, *P* < 0.01	Mean decrease: 40 ± 12% vs 41 ± 10% NS
		
		Ng *et al.* (2019)	Age 30.6 years, n = 199	Age 42.6 years, n = 242	10.6 years	Change from baseline, (pmol/l)	−13.5 ± 27.9, *P* < 0.001	–	–
	
	LH	Brown *et al.* (2011)	Age 28.6 years, n = 254	Age 29.9 years, n = 41	7 years	Median change per year, (mIU/ml)	<0.0001 (−0.0001 to 0.0001), NS	−0.45 (−0.75 to −0.15), *P* < 0.05	*P* < 0.001
		
		Carmina *et al.* (2012b)	Age 37 years, n = 54	Age 37 years, n = 20	5 years	Mean comparisons with baseline, (mUI/ml)	8.8 ± 4 vs 10 ± 3.7, NS	6.5 ± 1.1 vs 6.4 ± 1.4, NS	–
		
		Schmidt *et al.* (2011a)	Age 49.4 years, n = 25	Age 49.7 years, n = 68	21 years	Change from baseline, (IU/l)	11.2 ± 10.0, *P* = 0.001	7.4 ± 14.1, *P* = 0.001	*P* = 0.153
		
		Forslund *et al.* (2021)	Age 49.4 years, n = 21	Age 49.7 years, n = 55	32 years	mean change from baseline, (IU/l)	13.6 ± 9.5, *P* < 0.01	8.0 ± 13.2, *P* < 0.01	*P* = 0.22
		
		Ng *et al.* (2019)	Age 30.6 years, n = 199	Age 42.6 years, n = 242	10.6 years	Change from baseline, (IU/l)	−0.1 ± 11.3, *P* = 0.89	–	–
		
		Jarrett *et al.* (2020)	Age 26 years, n = 26	Age 30 years, n = 12	4 to 6 weeks	Over follicular and luteal phases, (mIU/ml)	Follicular phase: 13.1 ± 3.8 Luteal phase: 6.2 ± 3.3	Follicular phase: 9.8 ± 2.5 Luteal phase: 6.1 ± 2.8	Follicular phase: *P* = 0.02 Luteal phase: *P* = 0.95, All days: *P*_(PCOS)_ = 0.01
	FSH	Brown *et al.* (2011)	Age 28.6 years, n = 254	Age 29.9 years, n = 41	7 years	Median change per year, (mIU/ml)	0.18 (0.05, 0.32), *P* < 0.01	−0.59 (−0.98, −0.21), *P* < 0.05	*P* < 0.001
		
		Schmidt *et al.* (2011a)	Age 49.4 years, n = 25	Age 49.7 years, n = 68	21 years	Change from baseline, (U/l)	11.6 ± 38.0, *P* = 0.123	−13.9 ± 61.9, *P* = 0.12	*P* = 0.124
		
		Forslund *et al.* (2021)	Age 49.4 years, n = 21	Age 49.7 years, n = 55	32 years	Change from baseline, (IU/l)	24.9 ± 34.9, *P* = 0.03	2.7 ± 62, *P* = 0.73	*P* = 0.53
		
		Jarrett *et al.* (2020)	Age 26 years, n = 26	Age 30 years, n = 12	4 to 6 weeks	Over follicular and luteal phases, (mIU/ml)	Follicular phase: 6.0 ± 1.5 Luteal phase: 2.8 ± 1.2	Follicular phase: 7.8 ± 2.5 Luteal phase: 4.9 ± 2.0	Follicular phase: *P* = 0.05 Luteal phase: *P* < 0.01 All days: *P*_(PCOS)_ = 0.91
		
		Ng *et al.* (2019)	Age 30.6 years, n = 199	Age 42.6 years, n = 242	10.6 years	Change from baseline, (IU/l)	2.7 ± 8.0, *P* < 0.001	–	–
	
	LH:FSH ratio	Brown *et al.* (2011)	Age 28.6 years, n = 254	Age 29.9 years, n = 41	7 years	Median change per year	−0.06 (0.10 to −0.01), *P* < 0.05	<0.0001 (0.0001 to 0.0001), NS	*P* < 0.001
		
		Carmina *et al.* (2012b)	Age 37 years, n = 54	Age 37 years, n = 20	5 years	Mean comparisons with baseline	1.5 ± 0.7 vs 1.6 ± 0.7, NS	1 ± 0.3 vs 1.1 ± 0.2, NS	–
		
		Carmina *et al.* (2012a)	Age 21.9 years, n = 193	Not followed up	20 years	mean Comparisons (5th –20th years) with baseline	1.4 ± 0.5, 1.4 ± 0.6, 1.2 ± 0.4 vs 1.5 ± 0.6, NS	–	–
		
		Udesen *et al.* (2019)	Age 29.1 years, n = 40	Age 30.0 years, n = 8	5.8 years	Median comparison with baseline	1.7 (1.2–2.3) vs 1.6 (1.1–2.2), NS	0.7 (0.5–1.2) vs 0.8 (0.8–1.1), NS	–

Menstrual regularity		Brown *et al.* (2011)	Age 28.6 years, n = 254	Age 29.9 years, n = 41	7 years	% Regular cycles from baseline	4.6% vs 0%, *P* < 0.001	100% vs 100%, NS	*P* < 0.001
		
		Schmidt *et al.* (2011a)	Age 49.4 years, n = 25	Age 49.7 years, n = 68	21 years	Mean age of menopause (years)	50.1 ± 7.4	51.5 ± 4.8	*P* = 0.419

Chronic anovulation		–	–	–	–	–	–	–	–

Ovarian hyperstimulation syndrome		–	–	–	–	–	–	–	–

Pregnancy viability		–	–	–	–	–	–	–	–

A_4_, androstenedione; AMH, anti*-*Müllerian hormone; DHEAS, dehydroepiandrosterone sulphate; FAI, free androgen index; mFG, modified Ferriman-Gallwey score; NS, not significant; PCOS, polycystic ovary syndrome; SHBG, sex hormone-binding globulin; T, testosterone.

**Table III:** Pregnancy-related outcomes.

Outcomes	Study author, year	Baseline age, sample size		Mean follow-up duration	Effect measures	Observed estimates		
		PCOS group	Non-PCOS group			Within PCOS group comparison	Within non-PCOS group comparison	PCOS group versus non-PCOS group
Live births	–	–	–	–	–	–	–	–

Miscarriage	Palomba *et al.* (2014)	Age 27.8 years, n = 150, 5.4 gestational weeks	Age 27.4 years, n = 150 5.4 gestational week	27 gestational weeks	Cumulative rates	16.0%	5.3%	*P* = 0.004
	
	Jakubowicz *et al.* (2002)	Age 30 years, n = 31	–	4.5 years	Cumulative proportion (%)	41.9%	–	–
	
	Reyes-Munoz *et al.* (2012)	Age 29 years, n = 52	Age 29 years, n = 52	29.4 gestational weeks	Incidence proportion, RR	3.8%	1.9%	2.0 RR, 95% CI: 0.187–21.0

Stillbirths	Reyes-Munoz *et al.* (2012)	Age 29 years, n = 52	Age 29 years, n = 52	29.4 gestational weeks	Incidence proportion, RR	1.9%	3.8%	0.5 RR, 95% CI 0.04–5.3

Neonatal mortality	–	–	–	–	–	–	–	–

Gestational weight gain	Reyes-Munoz *et al.* (2012)	Age 29 years, n = 52	Age 29 years, n = 52	29.4 gestational weeks	Mean weight gain (kgs)	10.0 ± 5.4	10.8 ± 6.3	*P* = 0.513

Gestational diabetes	Palomba *et al.* (2014)	Age 27.8 years, n = 150, 5.4 gestational weeks	Age 27.4 years, n = 150 5.4 gestational weeks	27 gestational weeks	Cumulative rates	14.7%	5.3%	*P* = 0.011
	
	Reyes-Munoz *et al.* (2012)	Age 29 years, n = 52	Age 29 years, n = 52	29.4 gestational weeks	Incidence proportion, RR	26.9%	9.6%	2.8 RR, 95% CI 1.08–7.2

Preterm birth	Jakubowicz *et al.* (2002)	Age 30 years, n = 31	No controls	4.5 years	Cumulative proportion (%)	33.3%	–	–
	
	Reyes-Munoz *et al.* (2012)	Age 29 years, n = 52	Age 29 years, n = 52	29.4 gestational weeks	Incidence proportion, RR	11.5%	23.0%	0.5 RR, 95% CI 0.2–1.2

Hypertensive disease in pregnancy	Palomba *et al.* (2014)	Age 27.8 years, n = 150, 5.4 gestational weeks	Age 27.4 years, n = 150 5.4 gestational weeks	27 gestational weeks	Cumulative rates	12.7%	5.3%	*P* = 0.042
	
	Reyes-Munoz *et al.* (2012)	Age 29 years, n = 52	Age 29 years, n = 52	29.4 gestational weeks	Incidence proportion, RR	9.6%	11.5%	0.5 RR, 95%CI 0.27–2.5
Baby birth weight	Palomba *et al.* (2014)	Age 27.8 years, n = 150, 5.4 gestational weeks	Age 27.4 years, n = 150 5.4 gestational weeks	27 gestational weeks	Mean	3105.3 ± 346.1(g)	3179.8 ± 300.4 (g)	*P* = 0.039
	
	Reyes-Munoz *et al.* (2012)	Age 29 years, n = 52	Age 29 years, n = 52	29.4 gestational weeks	Mean	3,055 ± 552 (g)	2,976 ± 621 (g)	*P* = 0.513

Major congenital abnormalities	Reyes-Munoz *et al.* (2012)	Age 29 years, n = 52	Age 29 years, n = 52	29.4 gestational weeks	Incidence proportion	1.9%	1.9%	1.0 RR, 95% CI 0.06 to 15

kgs, kilograms; PCOS, polycystic ovary syndrome; RR, risk ratio.

**Table IV:** Psychologic and oncological outcomes.

Outcomes	Study author, year	Baseline age, sample size		Mean follow-up duration	Effect measures	Observed estimates		
		PCOS group	Non-PCOS group			Within PCOS group comparison	Within non-PCOS group comparison	PCOS group versus non-PCOS group
**Psychologic outcomes**								

Depression	Altinok *et al.* (2014)	Age 29 years, n = 1124	Age 29.0 years, n = 4213	PCOS: 6.8 years, controls: 7.2 years	Incidence proportions/HR (antidepressant prescription)	20%	15%	Versus population control: 0.75 HR (95% CI 0.64 to 0.88), *P* < 0.001 vs HTN control; *P* < 0.020
	
	Greenwood *et al.* (2019a)	Age 29 years, n = 163	No controls	5.5 years	BDI-FS score: median change from baseline Depression risk antidepressant use: % change	BDI-FS score: 0 (–2 to 1) enduring depression: 63% recovery: 37%	–	–
	
	Greenwood *et al.* (2019b)	Age 23 to 35 years, n = 83	Age 23 to 35 years, n = 1044	30 years at 5-year intervals	CES-D score change over lifetime	Score range: 11.2 to 13.4	Score range: 9 to 11.5	Coefficient, 2.51, 95% CI 1.49 to 3.54, *P* < 0.001
	
	Kerchner *et al.* (2009)	Age 32 years, n = 60	No controls	22 ± 3.7 months	Depression incidence and persistence from first survey	19% (incidence) 21.6% (persistence)	–	–

Anxiety	Harnod *et al.* (2020)	Age 27.7 years, n = 7026	Age 27.7 years, n = 28 104	16 years	Incidence rate and HR	15.3 per 1000	12.8 per 1000	1.18 HR 95% CI 1.07–1.30

Eating disorders	–	–	–	–	–	–	–	–

**Oncologic outcomes**								

Atypical hyperplasia	Cheng-Che *et al.* (2015)	Age 27 years, n = 3566	Age 7 years, n = 14 264	7.15 years	Incidence proportion/HR (breast cancer)	0.39%	0.21%	Cox: 1.98 HR (95% CI 1.03 to 3.80) Monte carlo: NS
	
	Schmidt *et al.* (2011b)	Age 49.4 ± 4.9 years, n = 25	Age 49.7 ± 5.6 years, n = 68	21 years	Incidence proportion (breast cancer)	9.4%	7.4%	NS

Endometrial cancer	Schmidt *et al.* (2011b)	Age 49.4 ± 4.9 years, n = 25	Age 49.7 ± 5.6 years, n = 68	21 years	Incidence proportion	0	0	–
	
	Cheng-Che *et al.* (2015)	Age 27 years, n = 3566	Age 7 years, n = 14 264	7.15 years	Incidence proportion/HR (uterine cancer)	0.14%	0.01%	8.4 HR 95%CI 1.6 to 43.9

BDI-FS, Beck Depression Inventory Fast Screen; CES-D, Center for Epidemiologic Studies-Depression; HR, hazard ratio; NS, not significant; PCOS, polycystic ovary syndrome.

### Diagnostic features and reproductive outcomes

Changes over time in all reproductive outcomes including clinical and biochemical hyperandrogenism, reproductive hormonal profiles and menstrual regularity are shown in Table II.

#### Clinical and biochemical hyperandrogenism

##### Hirsutism, acne and alopecia

Women with PCOS had significantly higher mFG scores than those without PCOS at 18 and 25 months of follow-up (Palomba *et al.*, 2007). However, the mFG score as a measure of hirsutism did not appear to change over time in women with or without PCOS, as indicated by two studies (Palomba *et al.*, 2007; Udesen *et al.*, 2019). None of the eligible studies reported longitudinal findings regarding acne or alopecia.

##### Testosterone

Ten studies reported data regarding changes in total T and three of these (Brown *et al.*, 2011; Schmidt *et al.*, 2011a; Forslund *et al.*, 2021) compared the total changes in T between women with and without PCOS. Two of the three studies (Brown *et al.*, 2011; Schmidt *et al.*, 2011a) indicated a significantly larger decline in total T over time among women with PCOS compared to those without PCOS, while one study showed (Forslund *et al.*, 2021) no significant difference in total T decline over time between the two groups. Of the seven studies that reported total T change from baseline within the PCOS group, five studies (Carmina *et al.*, 2012a,b; Huddleston *et al.*, 2017; Ng *et al.*, 2019; Udesen *et al.*, 2019) demonstrated significant declines, while one study (Palomba *et al.*, 2007) showed a non-significant increase. Another study among pregnant women with PCOS observed a significant increase in total T during gestation (Palomba *et al.*, 2007). Among women without PCOS, three studies indicated significant declines in T (Schmidt *et al.*, 2011a; Udesen *et al.*, 2019; Forslund *et al.*, 2021), while three studies did not observe a significant change (Palomba *et al.*, 2007; Carmina *et al.*, 2012a,b; Palomba *et al.*, 2014) in total T from baseline.

##### Free T and FAI

Udesen *et al.* (2019) observed significant declines in free T among women with PCOS, but not in those without PCOS. No other study assessed free T levels. Schmidt *et al.* (2011a) observed a significantly larger decline in FAI levels in women with PCOS than in women without PCOS over time, while the decline was not significantly different between the two groups in another study (Forslund *et al.*, 2021). Among studies that reported FAI changes in comparison with baseline within women with PCOS, two (Palomba *et al.*, 2007; Brown *et al.*, 2011) did not observe a significant change, while three studies in non-pregnant women (Schmidt *et al.*, 2011a; Ng *et al.*, 2019; Forslund *et al.*, 2021) did observe a change. One study in pregnant women (Palomba *et al.*, 2014) observed significant FAI declines. Among women without PCOS, two studies in non-pregnant women (Schmidt *et al.*, 2011a; Forslund *et al.*, 2021) and one study in pregnant women (Palomba *et al.*, 2014) indicated significant declines in FAI, although one study (Palomba *et al.*, 2007) did not observe a significant decline in FAI.

##### Dehydroepiandrosterone sulphate

All three studies that compared changes in DHEAS demonstrated significantly larger DHEAS declines among women with PCOS than women without (Brown *et al.*, 2011; Schmidt *et al.*, 2011a; Forslund *et al.*, 2021). Among women with PCOS, seven of the eight studies that compared DHEAS changes from baseline reported significant declines (Brown *et al.*, 2011; Schmidt *et al.*, 2011a; Carmina *et al.*, 2012a,b; Palomba *et al.*, 2014; Udesen *et al.*, 2019; Forslund *et al.*, 2021), but one study showed a non-significant decline (Palomba *et al.*, 2007). In women without PCOS, two studies indicated significant DHEAS declines (Schmidt *et al.*, 2011a; Forslund *et al.*, 2021), while five studies (Palomba *et al.*, 2007; Brown *et al.*, 2011; Carmina *et al.*, 2012b; Palomba *et al.*, 2014; Udesen *et al.*, 2019) indicated non-significant changes from baseline.

##### Androstenedione

Two studies compared changes in A_4_ between women with and without PCOS, with one study indicating a significantly larger decline in women with PCOS than the control group (Schmidt *et al.*, 2011a), and another study (Forslund *et al.*, 2021) showing that the rate of decline was similar between the two groups of women. Among women with PCOS, two studies (Brown *et al.*, 2011; Udesen *et al.*, 2019) revealed that A_4_ declined significantly from baseline, but four studies indicated no significant change (Palomba *et al.*, 2007; Schmidt *et al.*, 2011a; Palomba *et al.*, 2014; Forslund *et al.*, 2021). Among women without PCOS, one study demonstrated a significant A_4_ decline (Udesen *et al.*, 2019), but another study showed significant increases (Schmidt *et al.*, 2011a) and three studies (Palomba *et al.*, 2007, 2014; Forslund *et al.*, 2021) showed no significant change from baseline.

##### Sex hormone-binding globulin

Only two studies (Schmidt *et al.*, 2011a; Forslund *et al.*, 2021) compared SHBG changes between women with and without PCOS; both studies revealed that the change in SHBG was similar regardless of PCOS status. Among studies that reported SHBG change from baseline within PCOS women, two reported significant declines (Brown *et al.*, 2011; Udesen *et al.*, 2019), one observed a significant increase (Forslund *et al.*, 2021), and three did not observe a significant change in SHBG levels (Palomba *et al.*, 2007; Schmidt *et al.*, 2011a; Palomba *et al.*, 2014). In women without PCOS, three studies showed no significant differences over the time of the studies (Palomba *et al.*, 2007; 2014; Udesen *et al.*, 2019), although two reported significant SHBG increases compared to baseline (Udesen *et al.*, 2019; Forslund *et al.*, 2021).

#### Menstrual cycle regularity and chronic anovulation

One study demonstrated that women with PCOS had significantly fewer menstrual cycles per year compared to controls (Brown *et al.*, 2011), although a higher proportion of women with PCOS regained regular menstrual cycles over time, and the proportion of women regaining normal cycles did not change in non-PCOS women. In addition, age of menopause did not differ between women with and those without PCOS (Schmidt *et al.*, 2011a). We did not identify any longitudinal cohort studies that reported on the rate of chronic anovulation.

#### Other reproductive hormones

##### Anti-Müllerian hormone

Of the three studies that compared change in AMH, one study reported a significantly faster rate of AMH decline (Ahmad *et al.*, 2018) in women with PCOS than in women without, but two others reported no significant difference in change over time between the two groups of women (Brown *et al.*, 2011; Carmina *et al.*, 2012b). Among studies that reported changes in AMH levels over time within the PCOS group, two demonstrated a significant decline from baseline (Carmina *et al.*, 2012b; Ng *et al.*, 2019), while one showed no significant change (Brown *et al.*, 2011). Among women without PCOS, one study indicated no significant change in AMH levels from baseline (Brown *et al.*, 2011), while another study revealed significantly lower AMH levels at follow-up compared to baseline (Carmina *et al.*, 2012b).

##### LH, FSH and LH/FSH ratio

One study (Brown *et al.*, 2011) indicated that there was a more rapid decrease in LH levels per year in controls than in women with PCOS. However, two studies demonstrated that the mean change in LH over time was similar in women with and without PCOS (Schmidt *et al.*, 2011a; Forslund *et al.*, 2021). Furthermore, one study reported that LH was significantly higher across the follicular and luteal phases in women with anovulatory PCOS than in controls, while LH was significantly higher in women with PCOS who had sporadic ovulation compared to non-PCOS women, but only during the follicular phase and not in the luteal phase (Jarrett *et al.*, 2020). Within PCOS women, three studies showed no significant change in LH per year (Brown *et al.*, 2011) or from baseline (Carmina *et al.*, 2012b; Ng *et al.*, 2019), while two others indicated significant increases from baseline (Schmidt *et al.*, 2011a; Forslund *et al.*, 2021). Among women without PCOS, two studies showed significant increases in LH (Schmidt *et al.*, 2011a; Forslund *et al.*, 2021), but one indicated a significant decline per year (Brown *et al.*, 2011), and another showed no significant difference compared to baseline (Carmina *et al.*, 2012b).

One study reported that median FSH increased significantly in women with PCOS but decreased significantly in controls (Brown *et al.*, 2011). Jarrett *et al.* (2020), however, observed that FSH was significantly lower in women with PCOS and sporadic ovulation than in controls, although only during the luteal phase, but was similar between women with anovulatory PCOS and controls across both the luteal and follicular phases. Two studies (Schmidt *et al.*, 2011a; Forslund *et al.*, 2021) showed similar changes in FSH from baseline in women with and without PCOS. Finally, Ng *et al.* (2019) observed a significant increase in FSH from baseline in women with PCOS; data in women without PCOS was not reported.

Three studies indicated that the change in the LH/FSH ratio over time was similar in both women with PCOS and without PCOS (Carmina *et al.*, 2012a,b; Udesen *et al.*, 2019), although one study demonstrated a significant decrease in the median LH/FSH ratio per year in women with PCOS, but not in controls (Brown *et al.*, 2011).

### Pregnancy outcomes

Data related to pregnancy outcomes, including miscarriages, early pregnancy losses, stillbirth, pre-term birth, congenital malformations, gestational diabetes mellitus (GDM), hypertension in pregnancy-induced hypertension (PIH) and birthweight, are shown in Table III.

One study indicated that women with PCOS had a significantly higher cumulative rate of miscarriage than women without (Palomba *et al.*, 2014), but another study did not observe a significant difference in the incidence of miscarriage and stillbirths between women with and without PCOS (Reyes-Munoz *et al.*, 2012). One study did not observe a significant difference in the incidence of pre-term birth and congenital malformations between women with and without PCOS (Reyes-Munoz *et al.*, 2012). Two studies suggested that the incidence of GDM was significantly higher in women with PCOS compared to those without (Reyes-Munoz *et al.*, 2012; Palomba *et al.*, 2014). One study reported that the cumulative rates of PIH were significantly higher in women with PCOS than in controls (Palomba *et al.*, 2014), but another study did not observe a significant difference in the incidence of pre-eclampsia between women with and without PCOS (Reyes-Munoz *et al.*, 2012).

Palomba *et al.* (2014) reported that babies born to women with PCOS had significantly lower mean birth weight than those born to women without PCOS, although Reyes-Munoz *et al.* (2012) did not observe a significant difference in mean offspring birth weight between women with and without PCOS. Finally, there were no studies that met our inclusion criteria regarding live births and neonatal mortality in women with, compared to those without, PCOS.

### Oncological outcomes

One study indicated a similar incidence (0%) of endometrial cancer in both women with PCOS and controls (Schmidt *et al.*, 2011b), while another study (Cheng-Che *et al.*, 2015) showed that women with PCOS were eight times more likely to develop uterine cancer (presumably including endometrial cancer) compared to those without PCOS (Table IV). Furthermore, the same studies reported that the incidence rate of breast cancer was similar between women with and without PCOS during the follow-up period (Schmidt *et al.*, 2011b; Cheng-Che *et al.*, 2015).

### Psychological outcomes

As shown in Table IV, one Danish study reported a significantly higher incidence rate of anti-depressant medicine prescription in women with PCOS than in controls (Altinok *et al.*, 2014). Greenwood *et al.* (2019b) observed that the Center for Epidemiologic Studies-Depression (CES-D) scores in women with PCOS were two times higher compared to controls across the lifespan. However, another uncontrolled study among women with PCOS reported no significant change in the Beck Depression Inventory Fast Screen (BDI-FS) score and mood symptoms (Greenwood *et al.*, 2019a). Only one study reported that women with PCOS had an 18% higher risk of anxiety than those without PCOS (Harnod *et al.*, 2020). We did not identify eligible studies that reported on the incidence rate of eating disorders over time in women with and without PCOS.

## Discussion

This is the first comprehensive systematic review of longitudinal studies describing the natural history of reproductive, psychological and oncological outcomes in PCOS. It reports on outcomes aligned with the core outcomes set of recommended parameters that should be reported in PCOS studies (Al Wattar *et al.*, 2020).

### Diagnostic features and reproductive outcomes

This review demonstrates a uniform finding from two studies suggesting that clinical hyperandrogenism or mFG scores do not change significantly over time in either women with PCOS or those without PCOS (Palomba *et al.*, 2007; Udesen *et al.*, 2019). However, besides having the shortest average follow-up period (Supplementary Fig. S1), one of the studies (Palomba *et al.*, 2007) had a very limited sample size. Furthermore, mFG score varies by ethnicity (Javorsky *et al.*, 2014) and a large cross-sectional study has suggested that mFG scores may decline with age in the general population (Zhao *et al.*, 2011). Given the significant impact of hirsutism on quality of life (Teede *et al.*, 2018a,b), understanding the natural history of the disorder is important. Ethnic-specific evidence regarding mFG score changes across the life course are needed to provide insight into the natural history of hirsutism in PCOS and into how this may impact the diagnostic criteria of PCOS and the quality of life over time.

With respect to biochemical hyperandrogenism, SHBG, which impacts the free androgen status, was similar in women with and without PCOS. However, this evidence is from only two studies and participants in both studies were aged 49 years at baseline (Schmidt *et al.*, 2011a; Forslund *et al.*, 2021). Data are conflicting regarding total and free T, FAI and A_4_ between women with and without PCOS, which may be attributed to differences in participant characteristics and laboratory cutoffs or assays used. A significantly higher decline in DHEAS was noted among women with PCOS compared to those without PCOS, consistently reported by all three studies assessing this hormone (Brown *et al.*, 2011; Schmidt *et al.*, 2011a; Forslund *et al.*, 2021). Given that SHBG may be a potential biomarker for insulin resistance in PCOS (Deswal *et al.*, 2018), more research on the natural history evidence and impact on biochemical hyperandrogenism as a diagnostic feature in PCOS is needed.

Despite menstrual irregularity being one of the cardinal features of PCOS diagnosis (The Rotterdam ESHRE/ASRM-Sponsored PCOS Consensus Workshop Group, 2004; Teede *et al.*, 2018a), evidence assessing changes in menstrual cycle regularity over time (Brown *et al.*, 2011) was surprisingly insufficient in these cohort studies. This is consistent with the poor quality of data regarding menstrual cyclicity noted in the international guidelines (Teede *et al.*, 2018a,b).

Whether changes over time in other reproductive hormones, including LH, FSH and LH/FSH ratio, differ between women with and without PCOS is unclear, with conflicting findings and small numbers of relevant longitudinal studies. Findings from Jarrett *et al.* (2020) suggest that the levels of LH and FSH may differ significantly between women with and those without PCOS, depending on the phase of the menstrual cycle assessed. Similarly, evidence as to whether there are differences in the rate of decline in AMH concentrations over time in women with or without PCOS is inconclusive (Brown *et al.*, 2011; Carmina *et al.*, 2012b). In addition to differences in the AMH assays and cutoffs values used, circulating levels of AMH tend to vary across PCOS phenotypes (Rosenfield *et al.*, 2012; Teede *et al.*, 2019) and it is likely that there are phenotypic variations between participants, which may account for the conflicting results.

### Pregnancy outcomes

The risk of GDM appears to be higher in women with PCOS compared to those without PCOS as shown by two studies (Reyes-Munoz *et al.*, 2012; Palomba *et al.*, 2014). Although both studies were conducted in referred participants, this finding is consistent with the large body of literature from other systematic reviews of different study designs (Toulis *et al.*, 2009; Palomba *et al.*, 2015; Bahri Khomami *et al.*, 2019). The finding also aligns with current PCOS guidelines, which emphasize assessing pregnant women with PCOS for GDM (Teede *et al.*, 2018a,b). Overall, we currently lack data from homogenous prospective studies among medically unbiased populations to provide a comprehensive natural history of GDM in PCOS. This will enable identification of those most at risk for timely intervention and management. Evidence related to other pregnancy-related outcomes, including PIH, pre-eclampsia, birth weight, pre-term birth, congenital malformations and maternal weight gain is largely limited, which calls for further research.

### Oncological outcomes

Evidence as to whether the risk of endometrial or uterine cancer over time differs between women with and without PCOS is conflicting. Schmidt *et al.* (2011b) observed no significant difference in these risks, while Cheng-Che *et al.* (2015) observed a significantly higher risk in women with PCOS. However, both studies were methodologically limited, with a small overall sample sizes and numbers of incident cases. Moreover, endometrial alteration (Palomba *et al.*, 2021) and downregulation of various biological mechanisms in the endometrial stromal part (Kim *et al.*, 2009) occurs more among women with PCOS compared to those without PCOS. Furthermore, multiple risk factors, such as obesity, pre-existing hypertension and diabetes, anovulation, parity and family history modulate the relationship between PCOS and endometrial cancer (Navaratnarajah *et al.*, 2008) and studies that assess these factors are missing and hence there is a need for more evidence.

Similarly, additional longitudinal cohort studies assessing the risk of breast cancer between women with and without PCOS are needed, as the data are currently limited (Schmidt *et al.*, 2011b; Cheng-Che *et al.*, 2015).

### Psychological outcomes

Although anxiety and depression are key symptoms experienced by women with PCOS (Teede *et al.*, 2010; Tay *et al.*, 2019), longitudinal studies assessing these features are limited and/or include non-comparable endpoints.

### Strengths and limitations

In general, this review explores longitudinal changes in the reproductive, psychologic and oncologic features of PCOS over the life course. Our analysis focused on features and outcomes of PCOS as specified in the PCOS core outcomes set (Al Wattar *et al.*, 2020), including only studies where participants had a confirmed PCOS status according to the international guideline-recommended diagnostic criteria (Teede *et al.*, 2018a,b). The major limitation in the data observed is that most included studies are limited in numbers and time of follow-up, heterogeneous across age groups, and varied in study setting, ethnicity, follow-up duration, types of assays or tests used and effect measures. The insufficient number of studies for each outcome did not allow for meta-regression to further assess heterogeneity. The variations between studies also did not allow meta-analysis, differentiation between the various ethnic groups and the various phenotypes of PCOS. Another key limitation is that a substantial number of studies were uncontrolled (Jakubowicz *et al.*, 2002; Kerchner *et al.*, 2009; Carmina *et al.*, 2013; Greenwood *et al.*, 2019a) thus, their contribution was rather limited.

## Conclusion

Overall, our evidence synthesis indicates that there is still limited data that may be useful in exploring the long-term and natural history of PCOS and homogenous longitudinal studies reporting outcomes that are aligned with the PCOS core outcomes set are lacking. PCOS natural history related questions (Supplementary Table SIII) remain unanswered. Given the importance of understanding the natural history of PCOS, the need for long-term prospective cohort studies in well-profiled populations is paramount.

## Data availability

The data underlying this article are available in the article and in its online supplementary material.

## Authors’ roles

H.J.T., S.K. and A.E.J. conceptualized the study and drafted the protocol including the search strategy. S.K. performed the literature search and obtained full text copies of studies. S.K., C.T.T. and K.L.R. evaluated eligibility criteria. S.K. and K.L.R. extracted data, interpreted the results and drafted the manuscript, which was reviewed and approved by C.T.T., K.L.R., A.E.J., R.A. and H.J.T.

## Funding

This specific study was not funded. S.K. is supported by scholarships from the Research Training Program of the Commonwealth of Australia and Monash University; H.J.T. is supported by an Australian National Health and Medical Research Council fellowship; and A.E.J. is supported by the Australian National Health and Medical Research Council's Centre for Research Excellence in Women’s Health in Reproductive Life.

## Conflict of interest

R.A. was employed by the American Society for Reproductive Medicine and is a consultant to Spruce Biosciences and Fortress Biotech. All other authors have nothing to declare.

## Supplementary Material

deac077_Supplementary_Data_File_S1

deac077_Supplementary_Figure_S1

deac077_Supplementary_Table_SI

deac077_Supplementary_Table_SII

deac077_Supplementary_Table_SIII
