# Supplementary material for: Where are we in understanding the natural history of polycystic ovary syndrome? A systematic review of longitudinal cohort studies
Source: Hum Reprod. 2022 May 10;37(6):1255–73. doi: 10.1093/humrep/deac077 (PMC9206535; doi:10.1093/humrep/deac077)
Supplement: deac077_Supplementary_Data_File_S1 [file deac077_supplementary_data_file_s1.pdf]

Human Reproduction, pp. 1–5, 2022
https://doi.org/10.1093/humrep/deac077

human reproduction

SUPPLEMENTARY DATA

Supplementary Data File SI: Search strategy

EMBASE

Database: Embase Classic+Embase <1947 to 2021 February 09>
Search Strategy:
----

(1) polycystic ovary syndrome/(12519)
(2) polycystic ovar*.mp. (25615)
(3) PCO*.mp. (48927)
(4) anovulation/(6419)
(5) (anovulat* or oligoovulat*).mp. (10713)
(6) (ovar* adj5 (cyst or sclerocystic or polycystic or poly-cystic or degenerat* or hyperandrogen* or hyper-androgen*)).mp. (49126)
(7) or/1-6 (86252)
(8) disease progression/(97425)
(9) natural history.mp. or Natural History/(357568)
(10) puberty/or menarche/or sexual development/(60630)
(11) Pubarche.mp. (857)
(12) (Breast adj2 develop*).mp. [mp=title, abstract, heading word, drug trade name, original title, device manufacturer, drug manufacturer, device trade name, keyword, floating subheading word, candidate term word] (19065)
(13) thelarche.mp. (887)
(14) *medical records/or exp health records, personal/(268862)
(15) (medical and (record or history)).mp. [mp=title, abstract, heading word, drug trade name, original title, device manufacturer, drug manufacturer, device trade name, keyword, floating subheading word, candidate term word] (767228)
(16) or/8-15 (1241312)
(17) 7 and 16 (5292)
(18) ((progress* or indicator* or feature* or trait* or phenotyp* or spectrum or characteri*OR differentiat* or sequelae) adj2 (PCOS or polycystic)).mp. (2066)
(19) ((imbalance or change* or alteration* or course or manifestat* or development* or dysregulat* or etiolog* or progress* or pathogenesis or pathophysiolog*) adj3 (PCOS or polycystic)).mp. (3536)
(20) ((surveillance or longitudinal or long term or cohort) adj5 (PCOS or polycystic)).mp. (921)
(21) 18 or 19 or 20 (5718)
(22) 17 or 21 (10654)
(23) limit 22 to (humans and yr="1990 - Current") (9006)
(24) limit 23 to dc=20191001-20210210 (1272)

****

MEDLINE

Database: Ovid MEDLINE(R) <1946 to February 08, 2021>
Search Strategy:
----

(1) polycystic ovary syndrome/(14820)
(2) polycystic ovar*.mp. (17361)
(3) PCO*.mp. (28417)
(4) anovulation/(2218)
(5) (anovulat* or oligoovulat*).mp. (6062)
(6) (ovar* adj5 (cyst or sclerocystic or polycystic or poly-cystic or degenerat* or hyperandrogen* or hyper-androgen*)).mp. (21553)
(7) or/1-6 (43634)
(8) disease progression/(169969)
(9) natural history.mp. or Natural History/(44640)
(10) puberty/or menarche/or sexual development/(18599)
(11) Pubarche.mp. (445)
(12) (Breast adj2 develop*).mp. [mp=title, abstract, original title, name of substance word, subject heading word, floating subheading word, keyword heading word, organism supplementary concept word, protocol supplementary concept word, rare disease supplementary concept word, unique identifier, synonyms] (9778)
(13) thelarche.mp. (447)
(14) *medical records/or exp health records, personal/(47368)
(15) (medical and (record or history)).mp. [mp=title, abstract, original title, name of substance word, subject heading word, floating subheading word, keyword heading word, organism supplementary concept word, protocol supplementary concept word, rare disease supplementary concept word, unique identifier, synonyms] (201564)
(16) or/8-15 (475969)
(17) 7 and 16 (1122)
(18) ((progress* or indicator* or feature* or trait* or phenotyp* or spectrum or characteri*OR differentiat* or sequelae) adj2 (PCOS or polycystic)).mp. (1210)
(19) ((imbalance or change* or alteration* or course or manifestat* or development* or dysregulat* or etiolog* or progress* or pathogenesis or pathophysiolog*) adj3 (PCOS or polycystic)).mp. (2045)
(20) ((surveillance or longitudinal or long term or cohort) adj5 (PCOS or polycystic)).mp. (439)
(21) 18 or 19 or 20 (3239)
(22) 17 or 21 (4242)
(23) limit 22 to (humans and yr="1990 - Current") (3575)
(24) limit 23 to updaterange="pmfc(20191001-20210210)" (235)

****

psycINFO

Database: APA PsycInfo <1806 to February Week 1 2021>
Search Strategy:

---

(1) polycystic ovary syndrome/(0)
(2) polycystic ovar*.mp. (456)
(3) PCO*.mp. (1014)
(4) anovulation/(0)
(5) (anovulat* or oligoovulat*).mp. (155)
(6) (ovar* adj5 (cyst or sclerocystic or polycystic or poly-cystic or degenerat* or hyperandrogen* or hyper-androgen*)).mp. (471)
(7) or/1-6 (1350)
(8) disease progression/(1746)
(9) natural history.mp. or Natural History/(4223)
(10) puberty/or menarche/or sexual development/(4828)
(11) Pubarche.mp. (7)
(12) (Breast adj2 develop*).mp. [mp=title, abstract, heading word, table of contents, key concepts, original title, tests & measures, mesh] (478)
(13) thelarche.mp. (12)
(14) *medical records/or exp health records, personal/(2335)
(15) (medical and (record or history)).mp. [mp=title, abstract, heading word, table of contents, key concepts, original title, tests & measures, mesh] (235223)
(16) or/8-15 (245581)
(17) 7 and 16 (135)
(18) ((progress* or indicator* or feature* or trait* or phenotyp* or spectrum or characteri*OR differentiat* or sequelae) adj2 (PCOS or polycystic)).mp. (24)
(19) ((imbalance or change* or alteration* or course or manifestat* or development* or dysregulat* or etiolog* or progress* or pathogenesis or pathophysiolog*) adj3 (PCOS or polycystic)).mp. (44)
(20) ((surveillance or longitudinal or long term or cohort) adj5 (PCOS or polycystic)).mp. (7)
(21) 18 or 19 or 20 (67)
(22) 17 or 21 (193)
(23) limit 22 to (human and yr="1990 - 2021") (172)
(24) limit 23 to yr="2020 - 2021" (9)

****

Database: EBM Reviews—Cochrane Database of Systematic Reviews <2005 to January 28, 2021>, EBM Reviews—ACP Journal Club <1991 to January 2021>, EBM Reviews—Database of Abstracts of Reviews of Effects <1st Quarter 2016>, EBM Reviews—Cochrane Clinical Answers <January 2021>, EBM Reviews—Cochrane Central Register of Controlled Trials <January 2021>, EBM Reviews—Cochrane Methodology Register <3rd Quarter 2012>, EBM Reviews—Health Technology Assessment <4th Quarter 2016>, EBM Reviews—NHS Economic Evaluation Database <1st Quarter 2016>
Search Strategy:

---

(1) polycystic ovary syndrome/(1541)
(2) polycystic ovar*.mp. (4275)
(3) PCO*.mp. (5764)
(4) anovulation/(150)
(5) (anovulat* or oligoovulat*).mp. (1115)
(6) (ovar* adj5 (cyst or sclerocystic or polycystic or poly-cystic or degenerat* or hyperandrogen* or hyper-androgen*)).mp. (4860)
(7) or/1-6 (7961)
(8) disease progression/(7104)
(9) natural history.mp. or Natural History/(2914)
(10) puberty/or menarche/or sexual development/(369)
(11) Pubarche.mp. (33)
(12) (Breast adj2 develop*).mp. [mp=ti, ab, tx, kw, ct, ot, sh, hw] (875)
(13) thelarche.mp. (9)
(14) *medical records/or exp health records, personal/(218)
(15) (medical and (record or history)).mp. [mp=ti, ab, tx, kw, ct, ot, sh, hw] (73709)
(16) or/8-15 (84264)
(17) 7 and 16 (491)
(18) ((progress* or indicator* or feature* or trait* or phenotyp* or spectrum or characteri*OR differentiat* or sequelae) adj2 (PCOS or polycystic)).mp. (202)
(19) ((imbalance or change* or alteration* or course or manifestat* or development* or dysregulat* or etiolog* or progress* or pathogenesis or pathophysiolog*) adj3 (PCOS or polycystic)).mp. (380)
(20) ((surveillance or longitudinal or long term or cohort) adj5 (PCOS or polycystic)).mp. (137)
(21) 18 or 19 or 20 (627)
(22) 17 or 21 (1096)
(23) limit 22 to yr="1990 - 2021" (1064)
(24) limit 23 to humans (1063)
(25) limit 24 to yr="2020 - 2021" (171)

****

EBSCOhost

Wednesday, 10 February 2021, 7:13:55 p.m.

#	Query	Limiters/expanders	Last run via	Results
S26	S19 OR S23	Limiters—Publication Year: 2019–2021<br>Search modes—Boolean/Phrase	Interface—EBSCOhost Research Databases<br>Search Screen—Advanced<br>Search Database—CINAHL Plus	699
S25	S19 OR S23	Limiters—Publication Year: 1990–2021<br>Search modes—Boolean/Phrase	Interface—EBSCOhost Research Databases<br>Search Screen—Advanced<br>Search Database—CINAHL Plus	3759
S24	S19 OR S23	Search modes—Boolean/Phrase	Interface—EBSCOhost Research Databases<br>Search Screen—Advanced<br>Search Database—CINAHL Plus	3782
S23	S20 OR S21 OR S22	Search modes—Boolean/Phrase	Interface—EBSCOhost Research Databases<br>Search Screen—Advanced<br>Search Database—CINAHL Plus	1148
S22	(surveillance or longitudinal or long term or cohort) N5 (PCOS or	Search modes—Boolean/Phrase	Interface—EBSCOhost Research Databases<br>Search Screen—Advanced polycystic)<br>Search Database—CINAHL Plus	227
S21	(imbalance or change* or alteration* or course or manifestat* or development* or dysregulat* or etiolog* or progress* or pathogenesis or pathophysiolog*) N3 (PCOS or polycystic)	Search modes—Boolean/Phrase	Interface—EBSCOhost Research Databases<br>Search Screen—Advanced<br>Search Database—CINAHL Plus	712
S20	(progress* or indicator* or feature* or trait* or phenotyp* or spectrum or characteri*OR differentiat* or sequelae) N2 (PCOS or polycystic)	Search modes—Boolean/Phrase	Interface—EBSCOhost Research Databases<br>Search Screen—Advanced<br>Search Database—CINAHL Plus	395
S19	S7 AND S18	Search modes—Boolean/Phrase	Interface—EBSCOhost Research Databases<br>Search Screen—Advanced<br>Search Database—CINAHL Plus	2748
S18	S8 OR S9 OR S10 OR S11 OR S12 OR S13 OR S14 OR S15 OR S16 OR S17	Search modes—Boolean/Phrase	Interface—EBSCOhost Research Databases<br>Search Screen—Advanced<br>Search Database—CINAHL Plus	185 760
S17	medical and (record or history)	Search modes—Boolean/Phrase	Interface—EBSCOhost Research Databases<br>Search Screen—Advanced<br>Search Database—CINAHL Plus	126 275
S16	(MH "Medical Records")	Search modes—Boolean/Phrase	Interface—EBSCOhost Research Databases<br>Search Screen—Advanced<br>Search Database—CINAHL Plus	20 572

(continued)

Continued				
#	Query	Limiters/expanders	Last run via	Results
S15	thelarche	Search modes—Boolean/Phrase	Interface—EBSCOhost Research Databases<br>Search Screen—Advanced Search Database—CINAHL Plus	103
S14	"Breast N2 develop*"	Search modes—SmartText Searching	Interface—EBSCOhost Research Databases<br>Search Screen—Advanced Search Database—CINAHL Plus	16 269
S13	Pubarche	Search modes—Boolean/Phrase	Interface—EBSCOhost Research Databases<br>Search Screen—Advanced Search Database—CINAHL Plus	76
S12	"sexual development"	Search modes—Boolean/Phrase	Interface—EBSCOhost Research Databases<br>Search Screen—Advanced Search Database—CINAHL Plus	433
S11	(MH "Menarche")	Search modes—Boolean/Phrase	Interface—EBSCOhost Research Databases<br>Search Screen—Advanced Search Database—CINAHL Plus	1435
S10	(MH "Puberty")	Search modes—Boolean/Phrase	Interface—EBSCOhost Research Databases<br>Search Screen—Advanced Search Database—CINAHL Plus	3146
S9	"natural history"	Search modes—Boolean/Phrase	Interface—EBSCOhost Research Databases<br>Search Screen—Advanced Search Database—CINAHL Plus	9230
S8	(MH "Disease Progression")	Search modes—Boolean/Phrase	Interface—EBSCOhost Research Databases<br>Search Screen—Advanced Search Database—CINAHL Plus	48 674
S7	S1 OR S2 OR S3 OR S4 OR S5 OR S6	Search modes—Boolean/Phrase	Interface—EBSCOhost Research Databases<br>Search Screen—Advanced Search Database—CINAHL Plus	44 325
S6	ovar* N5 (cyst or sclerocystic or poly-cystic or poly-cystic or degenerat* or hyperandrogen* or hyperandrogen*)	Search modes—Boolean/Phrase	Interface—EBSCOhost Research Databases<br>Search Screen—Advanced Search Database—CINAHL Plus	41 543
S5	anovulat* or oligoovulat*	Search modes—Boolean/Phrase	Interface—EBSCOhost Research Databases<br>Search Screen—Advanced Search Database—CINAHL Plus	856
S4	(MH "Anovulation")	Search modes—Boolean/Phrase	Interface—EBSCOhost Research Databases<br>Search Screen—Advanced Search Database—CINAHL Plus	346
S3	"PCO*"	Search modes—Boolean/Phrase	Interface—EBSCOhost Research Databases<br>Search Screen—Advanced Search Database—CINAHL Plus	4954

(continued)

Continued

#	Query	Limiters/expanders	Last run via	Results
S2	"polycystic ovar*"	Search modes—Boolean/Phrase	Interface—EBSCOhost Research Databases<br>Search Screen—Advanced Search<br>Database—CINAHL Plus	5100
S1	(MH "Polycystic Ovary Syndrome")	Search modes—Boolean/Phrase	Interface—EBSCOhost Research Databases<br>Search Screen—Advanced Search<br>Database—CINAHL Plus	3984
