# Supplementary material for: Where are we in understanding the natural history of polycystic ovary syndrome? A systematic review of longitudinal cohort studies
Source: Hum Reprod. 2022 May 10;37(6):1255–73. doi: 10.1093/humrep/deac077 (PMC9206535; doi:10.1093/humrep/deac077)
Supplement: deac077_Supplementary_Figure_S1 [file deac077_supplementary_figure_s1.pdf]

Human Reproduction, pp. 1–1, 2022
https://doi.org/10.1093/humrep/deac077

human reproduction

SUPPLEMENTARY DATA

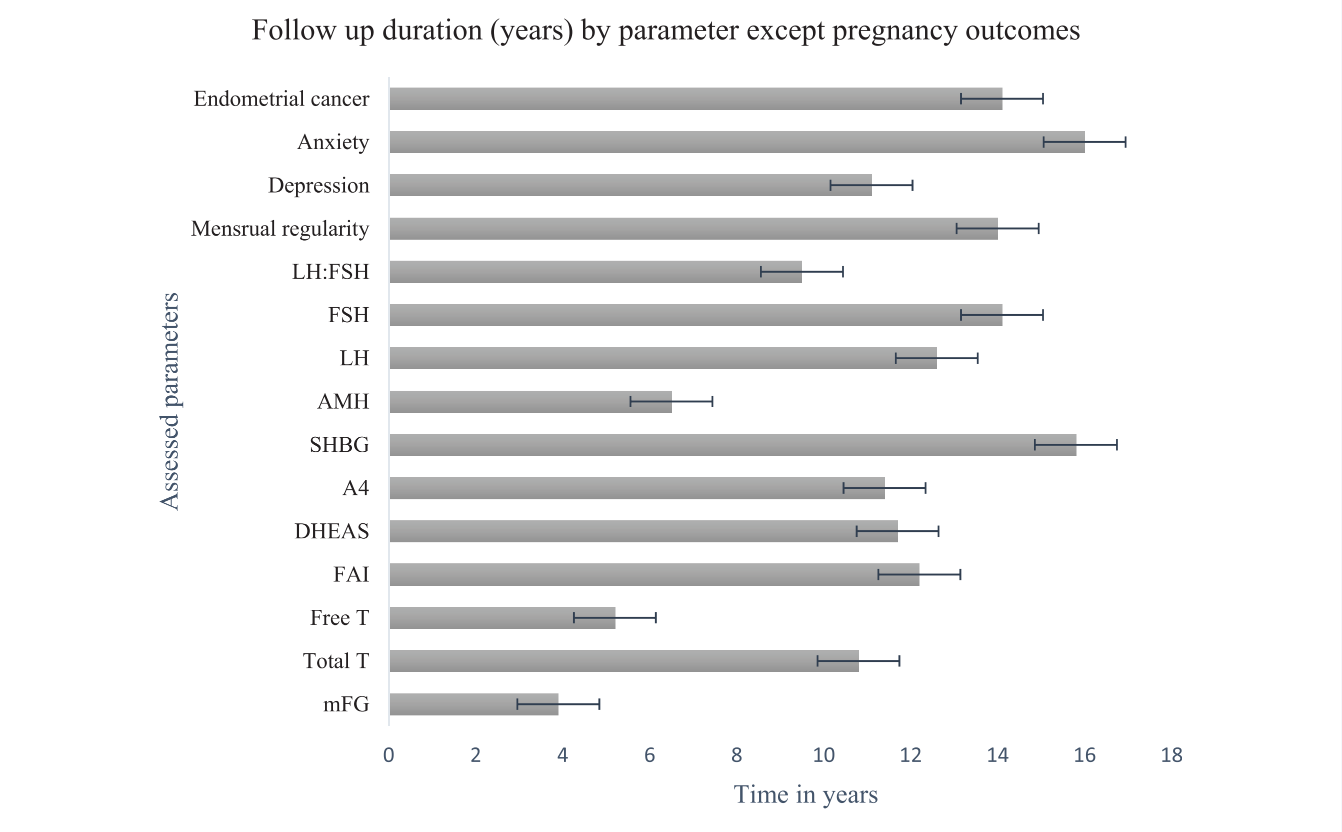

Supplementary Figure S1. Average follow-up duration for reproductive, psychological and oncological features. A4, androstenedione; AMH, anti-Müllerian hormone; DHEAS, dehydroepiandrosterone sulphate; FAI, free androgen index; mFG, modified Ferriman Gallwey score; SHBG, sex hormone-binding globulin; T, testosterone.
