# Supplementary material for: Where are we in understanding the natural history of polycystic ovary syndrome? A systematic review of longitudinal cohort studies
Source: Hum Reprod. 2022 May 10;37(6):1255–73. doi: 10.1093/humrep/deac077 (PMC9206535; doi:10.1093/humrep/deac077)
Supplement: deac077_Supplementary_Table_SI [file deac077_supplementary_table_si.pdf]

Human Reproduction, pp. 1–1, 2022
https://doi.org/10.1093/humrep/deac077

human reproduction

SUPPLEMENTARY DATA

Supplementary Table SI **Participant Intervention Comparison Outcomes and Studies (PICOS) framework.**

	Participants (P)	Intervention (I)	Comparison (C)	Outcomes (O)	Study type	Limits
**Inclusion criteria**	Female (all weight, lean or overweight) with PCOS as per Rotterdam consensus 2003 or NIH 1990	Follow-up (prospective or retrospective)	Female without PCOS (all weight, lean or overweight)	PCOS core outcomes<br>• Reproductive<br>• Metabolic<br>• Psychological<br>• Pregnancy outcomes<br>• Oncology related<br>• Adverse<br>• Long term (off-spring)	Cohort/longitudinal, cohort nested in RCT with non-PCOS arm	Publications from 1990 to date<br>Human studies
**Exclusion criteria**	Stein Leventhal syndrome<br>PCOS as per the Androgen Excess and PCOS society, 2006 criteria<br>On treatment such as IVF or pill				Cross-sectional studies<br>Editorial<br>Case reports<br>Commentaries<br>Narrative reviews<br>Expert opinion<br>Systematic reviews<br>Letters<br>Conference abstracts	Animal studies<br>Publications prior to 1990
