# Supplementary material for: Where are we in understanding the natural history of polycystic ovary syndrome? A systematic review of longitudinal cohort studies
Source: Hum Reprod. 2022 May 10;37(6):1255–73. doi: 10.1093/humrep/deac077 (PMC9206535; doi:10.1093/humrep/deac077)
Supplement: deac077_Supplementary_Table_SII [file deac077_supplementary_table_sii.pdf]

Human Reproduction, pp. 1–10, 2022
https://doi.org/10.1093/humrep/deac077

human reproduction

SUPPLEMENTARY DATA

Supplementary Table SII Articles excluded on full text.

Author	Title	Notes
**Abdel Gadir 1991**	Polycystic ovaries: do these represent a specific endocrinopathy?	Cross-sectional study/analysis
**Abdel-Rahman 2011**	Prevalence of abnormal glucose metabolism in a cohort of Arab women with polycystic ovary syndrome	Cross-sectional study/analysis
**Abedini 2019**	Zinc status and polycystic ovarian syndrome: a systematic review and meta-analysis	Systematic review—included studies ineligible
**Abruzzese 2015**	PCOS and metabolic disorders	Conference abstract
**Abu Hashim 2011**	Does laparoscopic ovarian diathermy change clomiphene-resistant PCOS into clomiphene-sensitive?	All are PCOS participants and received treatment/intervention
**Actrn 2011**	Vitamin D in women with polycystic ovary syndrome	Record of trial registration
**Actrn 2019**	Understanding the role of tissue Fibrosis in Insulin Resistance associated with Polycystic Ovary Syndrome (PCOS) and the impact of Exercise: the FIREx study using a cohort and randomized control trial in women with and without PCOS	Record of trial registration
**Adeniji 2016**	Metabolic effects of a commonly used combined hormonal oral contraceptive in women with and without polycystic ovary syndrome	All are PCOS participants and received treatment/intervention
**Alekseeva 1991**	[Pulsed secretion of gonadotropins in polycystic ovary syndrome]	All are PCOS participants and received treatment/intervention
**Amihaesei 2016**	Polycystic ovary syndrome, a complex entity involving reproductive and metabolic impairments: a systematic review	Narrative review
**Anderson 1998**	Inhibin A and inhibin B in women with polycystic ovarian syndrome during treatment with FSH to induce mono-ovulation	Inelegible PCOS definition criteria used
**Aroda 2009**	Metabolic and hormonal changes induced by pioglitazone in polycystic ovary syndrome: a randomized, placebo-controlled clinical trial	RCT, no non-PCOS arm
**Aye 2014**	Acute hypertriglyceridemia induces platelet hyperactivity that is not attenuated by insulin in polycystic ovary syndrome	All participants received treatment/intervention
**Bell 2018**	Maternal polycystic ovarian syndrome and early offspring development	PCOS definition criteria unclear/self-reported
**Bond 2017**	Diabetes risk in women with gestational diabetes mellitus and a history of polycystic ovary syndrome: a retrospective cohort study	PCOS definition criteria unclear/self-reported
**Bronstein 2011**	Age of onset of polycystic ovarian syndrome in girls may be earlier than previously thought	Wrong outcome
**Brown 2017**	Clomiphene and other antioestrogens for ovulation induction in polycystic ovarian syndrome	Systematic review included; studies ineligible
**Bruni 1991**	Natural contraceptive methods	Conference abstract
**Calan 2016**	A possible link between luteinizing hormone and macrophage migration inhibitory factor levels in polycystic ovary syndrome	Cross-sectional study/analysis
**Cappelli 2017**	Natural molecules for the therapy of hyperandrogenism and metabolic disorders in PCOS	Narrative review
**Cassidy-Vu 2016**	Role of statin drugs for polycystic ovary syndrome	Systematic review—included studies ineligible
**Centre for Reviews and Dissemination 2015**	What is the clinical effectiveness and cost-effectiveness of using drugs in treating obese patients in primary care? A systematic review (Structured abstract)	Not assessing PCOS

(continued)

Supplementary Table SII Continued

Author	Title	Notes
**Centre for Reviews and Dissemination 2015**	Use of metformin in polycystic ovary syndrome: a meta-analysis (Structured abstract)	Systematic review—included studies ineligible
**Centre for Reviews and Dissemination 2015**	Urinary hMG versus recombinant FSH for controlled ovarian hyper stimulation following an agonist long down-regulation protocol in IVF or ICSI treatment: a systematic review and meta-analysis (Structured abstract)	Not assessing PCOS
**Centre for Reviews and Dissemination 2015**	The role of metformin in polycystic ovary syndrome: a systematic review (Provisional abstract)	Systematic review—included studies ineligible
**Centre for Reviews and Dissemination 2015**	Systematic review and meta-analysis of the reproductive system effects of metformin alone or in combination with clomiphene citrate in patients with polycystic ovary syndrome (Provisional abstract)	Systematic review—included studies ineligible
**Centre for Reviews and Dissemination 2015**	Statin is a reasonable treatment option for patients with polycystic ovary syndrome: a meta-analysis of randomized controlled trials (Provisional abstract)	Systematic review—included studies ineligible
**Centre for Reviews and Dissemination 2015**	Rosiglitazone versus metformin for polycystic ovary syndrome: a systematic review (Provisional abstract)	Systematic review—included studies ineligible
**Centre for Reviews and Dissemination 2015**	N-acetylcysteine for treating women with clomiphene citrate resistant polycystic ovary syndrome: a systematic review (Provisional abstract)	Systematic review—included studies ineligible
**Centre for Reviews and Dissemination 2015**	Metformin vs thiazolidinediones for treatment of clinical, hormonal and metabolic characteristics of polycystic ovary syndrome: a meta-analysis (Structured abstract)	Systematic review—included studies ineligible
**Centre for Reviews and Dissemination 2015**	Metformin versus oral contraceptive pill in polycystic ovary syndrome: a Cochrane review (Structured abstract)	Systematic review—included studies ineligible
**Centre for Reviews and Dissemination 2015**	Metformin versus clomiphene citrate for infertility in non-obese women with polycystic ovary syndrome: a systematic review and meta-analysis (Provisional abstract)	Systematic review—included studies ineligible
**Centre for Reviews and Dissemination 2015**	Metformin in polycystic ovary syndrome: systematic review and meta-analysis (Structured abstract)	Systematic review—included studies ineligible
**Centre for Reviews and Dissemination 2015**	Metformin and gonadotropins for ovulation induction in patients with polycystic ovary syndrome: a systematic review with meta-analysis of randomized controlled trials (Provisional abstract)	Systematic review—included studies ineligible
**Centre for Reviews and Dissemination 2015**	Metabolomic biomarkers of impaired glucose tolerance and type 2 diabetes mellitus with a potential for risk stratification in women with polycystic ovary syndrome (Provisional abstract)	Systematic review—included studies ineligible
**Centre for Reviews and Dissemination 2015**	Meta-analysis of letrozole versus clomiphene citrate in polycystic ovary syndrome (Provisional abstract)	Systematic review—included studies ineligible
**Centre for Reviews and Dissemination 2015**	Lifestyle modification programs in polycystic ovary syndrome: systematic review and meta-analysis (Provisional abstract)	Systematic review—included studies ineligible
**Centre for Reviews and Dissemination 2015**	Letrozole for ovulation induction in women with polycystic ovarian syndrome: a systematic analysis (Provisional abstract)	Systematic review—included studies ineligible
**Centre for Reviews and Dissemination 2015**	Laparoscopic surgery versus laparotomy for women with polycystic ovarian syndrome: a systematic review (Provisional abstract)	Systematic review—included studies ineligible
**Centre for Reviews and Dissemination 2015**	Is a GnRH antagonist protocol better in PCOS patients? A meta-analysis of RCTs (Provisional abstract)	Systematic review—included studies ineligible
**Centre for Reviews and Dissemination 2015**	Exercise therapy in polycystic ovary syndrome: a systematic review (Provisional abstract)	Systematic review—included studies ineligible
**Centre for Reviews and Dissemination 2015**	Efficacy of inositol in women with polycystic ovary syndrome and desire for children: systematic review and meta-analysis (Provisional abstract)	Systematic review—included studies ineligible
**Centre for Reviews and Dissemination 2015**	Efficacy and safety of metformin during pregnancy in women with gestational diabetes mellitus or polycystic ovary syndrome: a systematic review (Provisional abstract)	Systematic review—included studies ineligible

(continued)

Supplementary Table SII Continued

Author	Title	Notes
**Centre for Reviews and Dissemination 2015**	Effects of thiazolidinediones on polycystic ovary syndrome: a meta-analysis of randomized placebo-controlled trials (Provisional abstract)	Systematic review—included studies ineligible
**Centre for Reviews and Dissemination 2015**	Effects of metformin in women with polycystic ovary syndrome treated with gonadotrophins for in vitro fertilisation and intracytoplasmic sperm injection cycles: a systematic review and meta-analysis of randomised controlled trials (Provisional abstract)	Systematic review—included studies ineligible
**Centre for Reviews and Dissemination 2015**	Effects of acarbose on polycystic ovary syndrome: a meta-analysis (Provisional abstract)	Systematic review—included studies ineligible
**Centre for Reviews and Dissemination 2015**	Effect of preconceptional metformin on abortion risk in polycystic ovary syndrome: a systematic review and meta-analysis of randomized controlled trials (Structured abstract)	Systematic review—included studies ineligible
**Centre for Reviews and Dissemination 2015**	Effect of metformin intervention during pregnancy on the gestational diabetes mellitus in women with polycystic ovary syndrome: a systematic review and meta-analysis (Provisional abstract)	Systematic review—included studies ineligible
**Centre for Reviews and Dissemination 2015**	Effect of lifestyle intervention on the reproductive endocrine profile in women with polycystic ovarian syndrome: a systematic review and meta-analysis (Provisional abstract)	Systematic review—included studies ineligible
**Centre for Reviews and Dissemination 2015**	Does metformin combined with clomiphene citrate improve fertility related outcomes in clomiphene resistant women with PCOS: a systematic review (Provisional abstract)	Systematic review—included studies ineligible
**Centre for Reviews and Dissemination 2015**	Dietary composition in the treatment of polycystic ovary syndrome: a systematic review to inform evidence-based guidelines (Structured abstract)	Systematic review—included studies ineligible
**Centre for Reviews and Dissemination 2015**	Comparative efficacy of thiazolidinediones and metformin for polycystic ovary syndrome (Provisional abstract)	Systematic review—included studies ineligible
**Centre for Reviews and Dissemination 2015**	Clomiphene citrate, metformin or both as first-step approach in treating anovulatory infertility in patients with polycystic ovary syndrome (PCOS): a systematic review of head-to-head randomized controlled studies and meta-analysis (Structured abstract)	Systematic review—included studies ineligible
**Centre for Reviews and Dissemination 2015**	Circulating markers of oxidative stress and polycystic ovary syndrome (PCOS): a systematic review and meta-analysis (Provisional abstract)	Systematic review—included studies ineligible
**Centre for Reviews and Dissemination 2015**	Can anti-Mullerian hormone predict the diagnosis of polycystic ovary syndrome? A systematic review and meta-analysis of extracted data (Provisional abstract)	Systematic review—included studies ineligible
**Centre for Reviews and Dissemination 2015**	Calpain-10 genetic polymorphisms and polycystic ovary syndrome risk: a meta-analysis and meta-regression (Provisional abstract)	Systematic review—included studies ineligible
**Centre for Reviews and Dissemination 2015**	Associations of adiponectin gene polymorphisms with polycystic ovary syndrome: a meta-analysis (Provisional abstract)	Systematic review—included studies ineligible
**Centre for Reviews and Dissemination 2015**	Association of Pro12Ala polymorphism in peroxisome proliferator-activated receptor gamma with polycystic ovary syndrome: a meta-analysis (Provisional abstract)	Systematic review—included studies ineligible
**Centre for Reviews and Dissemination 2015**	Aromatase inhibitors for PCOS: a systematic review and meta-analysis (Provisional abstract)	Systematic review—included studies ineligible
**Centre for Reviews and Dissemination 2015**	Aromatase inhibitors for infertility in polycystic ovary syndrome: the beginning or the end of a new era? (Structured abstract)	Systematic review—included studies ineligible
**Centre for Reviews and Dissemination 2015**	Adverse effects of the common treatments for polycystic ovary syndrome: a systematic review and meta-analysis (Provisional abstract)	Systematic review—included studies ineligible
**Centre for Reviews and Dissemination 2015**	A systematic review of the reproductive system effects of metformin in patients with polycystic ovary syndrome (Structured abstract)	Systematic review—included studies ineligible

(continued)

Supplementary Table SII Continued

Author	Title	Notes
**Centre for Reviews and Dissemination 2015**	A systematic review and meta-analysis of randomized controlled trials comparing pioglitazone versus metformin in the treatment of polycystic ovary syndrome (Provisional abstract)	Systematic review—included studies ineligible
**Centre for Reviews and Dissemination 2015**	A meta-analysis on acupuncture treatment of polycystic ovary syndrome (Provisional abstract)	Systematic review—included studies ineligible
**Centre for Reviews and Dissemination 2015**	A meta-analysis of polycystic ovary syndrome in women taking valproate for epilepsy (Provisional abstract)	Assessed non-PCOS population
**Chittenden 2009**	Polycystic ovary syndrome and the risk of gynaecological cancer: a systematic review	Systematic review—included studies ineligible
**Coffler 2003**	Evidence for abnormal granulosa cell responsiveness to follicle-stimulating hormone in women with polycystic ovary syndrome	PCOS participants received treatment/ intervention
**Dah-Ching 2018**	Association between polycystic ovarian syndrome and endometrial, ovarian, and breast cancer: a population-based cohort study in Taiwan	PCOS definition criteria unclear/used ICD-codes
**Dahlgren 1992**	Women with polycystic ovary syndrome wedge resected in 1956 to 1965: a long-term follow-up focusing on natural history and circulating hormones	PCOS participants received treatment/ intervention
**de Medeiros 2017**	Risks, benefits size and clinical implications of combined oral contraceptive use in women with polycystic ovary syndrome	Systematic review—included studies ineligible
**de Sousa 2012**	Longitudinal analyses of polysomnographic variables, serum androgens, and parameters of glucose metabolism in obese adolescents with polycystic ovarian syndrome	PCOS participants received treatment/ intervention
**El-Sharkawy 2014**	Effect of metformin on sleep disorders in adolescent girls with polycystic ovarian syndrome	Cross-sectional analysis
**Filippi 2017**	The risk of venous thromboembolism in women with polycistic ovarian syndrome: an Italian study	Conference abstract
**Finnbogadottir 2017**	Insulin resistance in pregnant women with and without polycystic ovary syndrome, and measures of body composition in offspring at birth and three years of age	Ineligible PCOS definition criteria used
**Forslund 2018**	Higher menopausal age but no differences in parity in women with polycystic ovary syndrome compared with controls	Cross-sectional study/analysis
**Forslund 2018**	Higher menopausal age but no differences in parity in women with polycystic ovary syndrome as compared to controls	Cross-sectional study/analysis
**Ganesan 2012**	Relationship between hormonal changes and folliculogenesis in polycystic ovarion syndrome women	Cross-sectional study/analysis
**Giallauria 2008**	Exercise training improves autonomic function and inflammatory pattern in women with polycystic ovary syndrome (PCOS)	RCT, no non-PCOS arm
**Glintborg 2015**	Morbidity and medicine prescriptions in a nationwide Danish population of patients diagnosed with polycystic ovary syndrome	Cross-sectional study/analysis
**Glintborg 2019**	Increased risk of thyroid disease in Danish women with polycystic ovary syndrome: a cohort study	PCOS definition criteria unclear/used ICD codes
**Glintborg 2018**	Cardiovascular disease in a nationwide population of Danish women with polycystic ovary syndrome	PCOS definition criteria unclear/used ICD codes
**Glueck 2011**	Sex hormone-binding globulin, oligomenorrhea, polycystic ovary syndrome, and childhood insulin at age 14 years predict metabolic syndrome and class III obesity at age 24 years	Cross-sectional study/analysis
**Glueck 2006**	Obesity, free testosterone, and cardiovascular risk factors in adolescents with polycystic ovary syndrome and regularly cycling adolescents	Cross-sectional study/analysis
**Gotby 2015**	Congenital Adrenal Hyperplasia, Polycystic Ovary Syndrome and criminal behavior: a Swedish population based study	Cross-sectional study/analysis
**Gottschau 2015**	Risk of cancer among women with polycystic ovary syndrome: a Danish cohort study	PCOS definition criteria unclear/used ICD codes

(continued)

Supplementary Table SII Continued

Author	Title	Notes
**Guraya 2017**	Correlation of clinical, radiological and serum analysis of hypovitaminosis D with polycystic ovary syndrome: a systematic review and meta-analysis	Systematic review—included studies ineligible
**Harnois-Leblanc 2017**	Evolution of metabolic alterations 5 years after early puberty in a cohort of girls predisposed to polycystic ovary syndrome	Inelegible PCOS definition criteria used
**Hart 2015**	The potential implications of a PCOS diagnosis on a woman's long-term health using data linkage	PCOS definition criteria unclear/used ICD codes
**Hillman 2014**	Black women with polycystic ovary syndrome (PCOS) have increased risk for metabolic syndrome and cardiovascular disease compared with white women with PCOS [corrected]	Cross-sectional study/analysis
**Hoeger 2004**	Review: Metformin used alone or combined with clomifene may improve ovulation rates in the polycystic ovary syndrome	Commentary
**Homburg 2017**	A novel method to demonstrate that pregnant women with polycystic ovary syndrome hyper-expose their fetus to androgens as a possible stepping-stone for the developmental theory of PCOS. A pilot study	Ineligible outcome
**Hudecova 2011**	Androgen levels, insulin sensitivity, and early insulin response in women with polycystic ovary syndrome: a long-term follow-up study	PCOS definition criteria unclear/self-reported
**Hudecova 2011**	Diabetes and impaired glucose tolerance in patients with polycystic ovary syndrome—a long term follow-up	Cross-sectional study/analysis
**Hudecova 2011**	Prevalence of the metabolic syndrome in women with a previous diagnosis of polycystic ovary syndrome: long-term follow-up	Cross-sectional study/analysis
**Hudecova 2010**	Endothelial function in patients with polycystic ovary syndrome: a long-term follow-up study	Cross-sectional study/analysis
**Hung 2014**	Risk of psychiatric disorders following polycystic ovary syndrome: a nationwide population-based cohort study	PCOS definition criteria unclear/used-ICD codes
**Irani 2014**	Vitamin D increases serum levels of the soluble receptor for advanced glycation end products in women with PCOS	PCOS participants received treatment/intervention
**Jacewicz-Swiecka 2018**	Polycystic ovary syndrome and the risk of cardio metabolic complications in longitudinal studies	Systematic review—included studies ineligible
**Jonsdottir 2017**	Obstetrical complications in dichorionic twin pregnancies in women with polycystic ovary syndrome	Cross-sectional study/analysis
**Klevedal 2017**	Fetal-maternal outcomes and complications in pregnant women with polycystic ovary syndrome	Cross-sectional study/analysis
**Krentz 2009**	Reduced serum ghrelin in a putative postmenopausal polycystic ovary syndrome phenotype	Cross-sectional study/analysis
**Kruszynska 2014**	Proinsulin, adiponectin and hsCRP in reproductive age women with polycystic ovary syndrome (PCOS)—the effect of metformin treatment	RCT, no non-PCOS arm
**Kumarendran 2018**	Polycystic ovary syndrome, androgen excess, and the risk of nonalcoholic fatty liver disease in women: a longitudinal study based on a United Kingdom primary care database	PCOS definition criteria unclear/used-ICD codes
**Kupesic 1996**	Polycystic ovary syndrome—Doppler parameters	Ineligible outcome
**Legro 2017**	Normal pubertal development in daughters of women With PCOS: a controlled study	Cross-sectional study/analysis
**Legro 2016**	Normal pubertal development in daughters of women with PCOS: a controlled study	Cross-sectional study/analysis
**Liao 2019**	Integrative Chinese herbal medicine therapy reduced the risk of type 2 diabetes mellitus in patients with polycystic ovary syndrome: a nationwide matched cohort study	PCOS definition criteria unclear/used ICD codes
**Lin 2017**	Risk of developing obstructive sleep apnea among women with polycystic ovarian syndrome: a nationwide longitudinal follow-up study	PCOS definition criteria unclear/used ICD codes

(continued)

Supplementary Table SII Continued

Author	Title	Notes
**Lord 2003**	Insulin-sensitising drugs (metformin, troglitazone, rosiglitazone, pioglitazone, D-chiro-inositol) for polycystic ovary syndrome	Systematic review—included studies ineligible
**Majkowska 1993**	[Insulin resistance and hyperinsulinemia in pathogenesis of polycystic ovary syndrome]	Narrative review
**Maliqueo 2009**	Adrenal function during childhood and puberty in daughters of women with polycystic ovary syndrome	Cross-sectional study/analysis
**Mani 2013**	Diabetes and cardiovascular events in women with polycystic ovary syndrome: a 20-year retrospective cohort study	PCOS definition criteria unclear
**Merz 2016**	Cardiovascular disease and 10-year mortality in postmenopausal women with clinical features of polycystic ovary syndrome	PCOS definition criteria unclear/self-reported
**Morgan 2012**	Evaluation of adverse outcome in young women with polycystic ovary syndrome versus matched, reference controls: a retrospective, observational study	PCOS definition criteria unclear
**Mumm 2015**	Hyperandrogenism and phenotypes of polycystic ovary syndrome are not associated with differences in obstetric outcomes	Cross-sectional study/analysis
**Naver 2014**	Increased risk of preterm delivery and pre-eclampsia in women with polycystic ovary syndrome and hyperandrogenaemia	Cross-sectional study/analysis
**Norman 2001**	Relative risk of conversion from normoglycaemia to impaired glucose tolerance or non-insulin dependent diabetes mellitus in polycystic ovarian syndrome	Ineligible PCOS definition criteria
**NunezRodriguez 2016**	Endocrine metabolic disorders of the dermatologic manifestation in the polycystic ovary syndrome	Cross-sectional study/analysis
**Ollila 2016**	Weight gain and dyslipidemia in early adulthood associate with polycystic ovary syndrome: prospective cohort study	PCOS definition criteria unclear/self-reported
**Ollila 2017**	Overweight and obese but not normal weight women with PCOS are at increased risk of Type 2 diabetes mellitus-a prospective, population-based cohort study	PCOS definition criteria unclear/self-reported
**Palomba 2015**	Pregnancy complications in women with polycystic ovary syndrome	Narrative review
**Palomba 2014**	Early trophoblast invasion and placentation in women with different PCOS phenotypes	Ineligible outcome
**Palomba 2010**	Pregnancy in women with polycystic ovary syndrome: the effect of different phenotypes and features on obstetric and neonatal outcomes	Cross-sectional study/analysis
**Palomba 2012**	Pervasive developmental disorders in children of hyperandrogenic women with polycystic ovary syndrome: a longitudinal case-control study	Cross-sectional study/analysis
**Paoletti 1996**	The chronic administration of cabergoline normalizes androgen secretion and improves menstrual cyclicity in women with polycystic ovary syndrome	Cross-sectional study/analysis
**Paoletti 1999**	Treatment with flutamide improves hyperinsulinemia in women with idiopathic hirsutism	RCT, non-PCOS arm not followed up
**Pasquali 2000**	Effect of long-term treatment with metformin added to hypocaloric diet on body composition, fat distribution, and androgen and insulin levels in abdominally obese women with and without the polycystic ovary syndrome	PCOS participants received treatment/intervention
**Piaditis 1996**	The effect of sequential administration of octreotide alone and octreotide/growth hormone simultaneously on buserelin stimulated ovarian steroid secretion in women with polycystic ovary syndrome	Ineligible PCOS definition criteria used
**Pierpoint 1998**	Mortality of women with polycystic ovary syndrome at long-term follow-up	PCOS definition criteria unclear

(continued)

Supplementary Table SII Continued

Author	Title	Notes
**Polotsky 2014**	Hyperandrogenic oligomenorrhea and metabolic risks across menopausal transition	Not assessing PCOS
**Pontikis 2011**	The incretin effect and secretion in obese and lean women with polycystic ovary syndrome: a pilot study	Cross-sectional study/analysis
**Poretsky 1994**	Insulin resistance, hypersecretion of LH, and a dual-defect hypothesis for the pathogenesis of polycystic ovary syndrome	Narrative review
**Prelevic 1990**	LH pulsatility and response to a single s.c. injection of buserelin in polycystic ovary syndrome	Inelegible PCOS definition criteria used
**Renke 2013**	Sonographic evaluation of the endometrium: monitoring progression of polycystic ovary syndrome. [German]	Case report
**Robin 2005**	Nutritional disorders in polycystic ovary syndrome: pathophysiological implications and therapeutics	Narrative review
**Roos 2011**	Risk of adverse pregnancy outcomes in women with polycystic ovary syndrome: population based cohort study	Inelegible PCOS definition criteria used
**Rosenfield 2015**	Adolescent polycystic ovary syndrome due to functional ovarian hyperandrogenism persists into adulthood	Inelegible PCOS definition criteria used
**Rubin 2017**	Development and risk factors of type 2 diabetes in a nationwide population of women with polycystic ovary syndrome	PCOS definition criteria unclear/used ICD codes
**Sadeghpoor 2017**	The possibility and management sterateghies of pregnancy in women with polycystic ovary syndrome: a review article	Conference abstract
**Sanchez-Santos 2018**	Prevalence and incidence of clinically diagnosed knee, hip and hand osteoarthritis in women with polycystic ovary syndrome: a national register-based study	Conference abstract
**Schmidt 2012**	Body composition, bone mineral density and fractures in late postmenopausal women with polycystic ovary syndrome—a long-term follow-up study	Similar results of interest in previous article (Schmidt 2011, #7100) by the same Author
**Sharpe 2019**	Metformin for ovulation induction (excluding gonadotrophins) in women with polycystic ovary syndrome	Systematic review—included studies ineligible
**Shaw 2008**	Postmenopausal women with a history of irregular menses and elevated androgen measurements at high risk for worsening cardiovascular event-free survival: results from the National Institutes of Health—National Heart, Lung, and Blood Institute sponsored Women's Ischemia Syndrome Evaluation	Retracted/withdrawn by author
**Shojaeian 2019**	Calcium and Vitamin D supplementation effects on metabolic factors, menstrual cycles and follicular responses in women with polycystic ovary syndrome: a systematic review and meta-analysis	Systematic review—included studies ineligible
**Stassek 2015**	Do pregnancy and parenthood affect the course of PCO syndrome? Initial results from the LIPCOS Study (Lifestyle Intervention for Patients with Polycystic Ovary Syndrome [PCOS])	Cross-sectional study/analysis
**Steck 1991**	[Is increased secretion of LH after LHRH administration a reliable indicator of polycystic ovary syndrome?]	Cross-sectional study/analysis
**Szosland 2015**	Prolactin secretion in polycystic ovary syndrome (PCOS)	Cross-sectional study/analysis
**Tang 2013**	Use of metformin for women with polycystic ovary syndrome to improve metabolic parameters	Systematic review—included studies ineligible
**Tang 2012**	Insulin-sensitising drugs (metformin, rosiglitazone, pioglitazone, D-chiro-inositol) for women with polycystic ovary syndrome, oligo amenorrhoea and subfertility	Systematic review—included studies ineligible
**Tang 2010**	Insulin-sensitising drugs (metformin, rosiglitazone, pioglitazone, D-chiro-inositol) for women with polycystic ovary syndrome, oligo amenorrhoea and subfertility	Systematic review—included studies ineligible
**Tang 2009**	Insulin-sensitising drugs (metformin, rosiglitazone, pioglitazone, D-chiro-inositol) for women with polycystic ovary syndrome, oligo amenorrhoea and subfertility	Systematic review/meta-analysis—included studies ineligible

(continued)

Supplementary Table SII Continued

Author	Title	Notes
**Thethi 2015**	Role of insulin sensitizers on cardiovascular risk factors in polycystic ovarian syndrome: a meta-analysis	Systematic review/meta-analysis—included studies ineligible
**Toscani 2011**	Effect of high-protein or normal-protein diet on weight loss, body composition, hormone, and metabolic profile in southern Brazilian women with polycystic ovary syndrome: a randomized study	All participants received treatment/intervention
**Trimeche 2004**	[Polycystic ovary syndrome in pubertal period: clinical, biological, metabolic and genetic polymorphism]	Cross-sectional study/analysis
**Tsikouras 2015**	Features of polycystic ovary syndrome in adolescence	Narrative review
**Underdal 2019**	Impaired respiratory function in women with PCOS compared to matched controls from a population-based study	Cross-sectional study/analysis
**Underdal 2019**	Prolactin and breast increase during pregnancy in PCOS: linked to long-term metabolic health?	Cross-sectional study/analysis
**Underdal 2017**	Metabolic health in women with PCOS-5-11 years' followup after metformin or placebo in pregnancy	Conference abstract
**Vasiljevic 2000**	[The role of insulin and hyperinsulinemia in the pathogenesis of the polycystic ovary syndrome]	Narrative review
**Vassalou 2019**	PCOS diagnosis in adolescents: the timeline of a controversy in a systematic review	Systematic review—included studies ineligible
**Veldhuis 2001**	Disruption of the synchronous secretion of leptin, LH, and ovarian androgens in nonobese adolescents with the polycystic ovarian syndrome	Cross-sectional study/analysis
**Veltman-Verhulst 2012**	Emotional distress is a common risk in women with polycystic ovary syndrome: a systematic review and meta-analysis of 28 studies	Systematic review—included studies ineligible
**Veras 2011**	Sexual dysfunction in patients with polycystic ovary syndrome: clinical and hormonal correlations	Cross-sectional study/analysis
**Vytiska-Binstorfer 1992**	[The endocrine status of young girls]	Not assessing PCOS
**Wang 2018**	Higher circulating irisin levels in patients with polycystic ovary syndrome: a meta-analysis	Meta-analysis; included studies ineligible
**Wang 2018**	A potential link between polycystic ovary syndrome and non-alcoholic fatty liver disease: an update meta-analysis	Meta-analysis; included studies ineligible
**Wang 2010**	Different phenotypes of polycystic ovary syndrome by Rotterdam criteria are differently steroidogenic but similarly insulin resistant	Cross-sectional study/analysis
**Wild 2002**	Long-term health consequences of PCOS	Narrative review
**Wild 2000**	Cardiovascular disease in women with polycystic ovary syndrome at long-term follow-up: a retrospective cohort study	PCOS definition criteria unclear
**Wise 2007**	Polycystic ovary syndrome and risk of uterine leiomyomata	PCOS definition criteria ineligible/self-reported
**Wiser 2013**	Age-related normogram for antral follicle count in women with polycystic ovary syndrome	Cross-sectional study/analysis
**Wu 2017**	5alpha-reductase activity in women with polycystic ovary syndrome: a systematic review and meta-analysis	Systematic review—included studies ineligible
**Wu 2016**	Acupuncture for treating polycystic ovary syndrome: guidance for future randomized controlled trials	Systematic review—included studies ineligible
**Xue 2017**	Effect of vitamin D on biochemical parameters in polycystic ovary syndrome women: a meta-analysis	Systematic review—included studies ineligible
**Yang 2018**	Increased risk of fractures in patients with polycystic ovary syndrome: a nationwide population-based retrospective cohort study	Ineligible outcome and unclear PCOS criteria
**Yang 2015**	Efficacy and safety of metformin or oral contraceptives, or both in polycystic ovary syndrome	Systematic review—included studies ineligible
**Yildiz 2008**	Impact of obesity on the risk for polycystic ovary syndrome	Cross-sectional study/analysis

(continued)

Supplementary Table SII Continued

Author	Title	Notes
**Zhou 2017**	Association between polycystic ovary syndrome and the risk of stroke and all-cause mortality: insights from a meta-analysis	Systematic review/meta-analysis—included studies ineligible
**Anonymous author, 2004**	Prevention of progression from precocious pubarche to polycystic ovary syndrome	Notes
**Manvelyan 2020**	The impact of polycystic ovarian syndrome on evaluation of reproductive potential measured by anti-muellerian and follicle stimulating hormones	Conference abstract
**Gunn 2019**	Polycystic ovarian syndrome in adolescents: utilising discovery proteomics and the search for to identify novel non-invasive biomarkers	Conference abstract
**deMedeiros 2020**	Changes in clinical and biochemical characteristics of polycystic ovary syndrome with advancing age	Cross-sectional study/analysis
**Lee 2021**	Postpartum weight retention in women with polycystic ovary syndrome	PCOS definition criteria unclear/self-reported
**Andersen 2019**	Third trimester cortisol status is associated with offspring sex and polycystic ovary syndrome status: Odense Child Cohort	Ineligible outcome
**Mills 2020**	Polycystic ovary syndrome as an independent risk factor for gestational diabetes and hypertensive disorders of pregnancy: a population-based study on 9.1 million pregnancies	PCOS definition criteria unclear/self-reported
**Robinson 2020**	The associations of maternal polycystic ovary syndrome and hirsutism with behavioral problems in offspring	PCOS definition criteria unclear/self-reported
**BahriKhomami 2019**	Lifestyle and pregnancy complications in polycystic ovary syndrome: the SCOPE cohort study	PCOS definition criteria unclear/self-reported
**Benson 2020**	Depression in girls with obesity and polycystic ovary syndrome and/or type 2 diabetes	Cross-sectional study/analysis
**Zhu 2021**	Polycystic ovary syndrome and risk of type 2 diabetes, coronary heart disease, and stroke	Ineligible design/not cohort
**Chen 2020**	Risk of bipolar disorder in patients with polycystic ovary syndrome: a nationwide population-based cohort study	PCOS definition criteria unclear/self-reported
**Lee 2020**	HIGHER RISK OF PERSISTENT METABOLIC SYNDROME (METSYN) IN BLACK WOMEN WITH POLYCYSTIC OVARY SYNDROME (PCOS): A LONGITUDINAL STUDY	Conference abstract
**Tadaion Far 2019**	Comparison of the umbilical cord Blood's anti-Mullerian hormone level in the newborns of mothers with polycystic ovary syndrome (PCOS) and healthy mothers	Cross-sectional study/analysis
**Tobiasz 2020**	Lack of fetal insulin resistance in maternal polycystic ovary syndrome	Cross-sectional study/analysis
**Wu 2020**	Hypertension risk in young women with polycystic ovary syndrome: a nationwide population-based cohort study	PCOS definition criteria unclear/self-reported
**Lee 2020**	Higher risk of persistent metabolic syndrome (Metsyn) in Black women with polycystic ovary syndrome (PCOS): a longitudinal study	Conference abstract/duplicate
**Komlosi 2019**	Pih6 metabolics syndrome and complications pregnant with PCOS	Conference abstract
**Helvaci 2020**	Cardiovascular health and menopause in aging women with polycystic ovary syndrome	Systematic review of different study designs-included studies don't meet our criteria
**Underdal 2020**	Impaired respiratory function in women with PCOS compared with matched controls from a population-based study	Cross-sectional study/analysis
**Underdal 2019**	Impaired respiratory function in women with PCOS compared with matched controls from a population-based study	Cross-sectional study/analysis
**Carmina 2019**	Characterization of metabolic changes in the phenotypes of women with polycystic ovary syndrome in a large Mediterranean population from Sicily	Cross-sectional study/analysis
**Harnod 2019**	Association between depression risk and polycystic ovarian syndrome in young women: a retrospective nationwide population-based cohort study (1998-2013)	PCOS definition criteria unclear/self-reported

(continued)

Supplementary Table SII Continued

Author	Title	Notes
**Feichtinger 2021**	Maternal overweight vs. polycystic ovary syndrome: disentangling their impact on insulin action in pregnancy-a prospective study	Cross-sectional study/analysis
**Valdimarsdottir 2019**	Pregnancy and neonatal complications in women with polycystic ovary syndrome in relation to second-trimester anti-Mullerian hormone levels	Cross-sectional study/analysis
**TadaionFar 2019**	Comparison of the umbilical cord Blood's anti-Mullerian hormone level in the newborns of mothers with polycystic ovary syndrome (PCOS) and healthy mothers	Cross-sectional study/analysis
**Zhang 2020**	Fetal growth, fetal development, and placental features in women with polycystic ovary syndrome: analysis based on fetal and placental magnetic resonance imaging	PCOS definition criteria unclear/self-reported
**Koivuaho 2019**	Age at adiposity rebound in childhood is associated with PCOS diagnosis and obesity in adulthood-longitudinal analysis of BMI data from birth to age 46 in cases of PCOS	PCOS definition criteria unclear/self-reported
**Kazemi 2019**	Comprehensive evaluation of type 2 diabetes and cardiovascular disease risk profiles in reproductive-age women with polycystic ovary syndrome: a large canadian cohort	Cross-sectional study/analysis
**Dahlgren 2019**	Reprint of: Women with polycystic ovary syndrome wedge resected in 1956 to 1965: a long-term follow-up focusing on natural history and circulating hormones	PCOS definition criteria unclear/self-reported
**Zhu 2020**	Polycystic ovary syndrome and risk of type 2 diabetes, coronary heart disease, and stroke	Ineligible design/not cohort
**Foroozanfard 2020**	Comparing pregnancy, childbirth, and neonatal outcomes in women with different phenotypes of polycystic ovary syndrome and healthy women: a prospective cohort study	PCOS group received treatment/intervention
**Overgaard 2020**	Maternal prolactin is associated with glucose status and PCOS in pregnancy: Odense Child Cohort	PCOS definition criteria unclear/self-reported
**Meun 2018**	High androgens in postmenopausal women and the risk for atherosclerosis and cardiovascular disease: the Rotterdam Study	Wrong PCOS definition criteria used
**Persson 2019**	Fecundity among women with polycystic ovary syndrome (PCOS)-A population-based study	PCOS definition criteria unclear/self-reported
**Behboudi-Gandevani 2020**	The risk of chronic kidney disease among women with polycystic ovary syndrome: a long-term population-based cohort study	Ineligible outcome
