# Supplementary material for: Where are we in understanding the natural history of polycystic ovary syndrome? A systematic review of longitudinal cohort studies
Source: Hum Reprod. 2022 May 10;37(6):1255–73. doi: 10.1093/humrep/deac077 (PMC9206535; doi:10.1093/humrep/deac077)
Supplement: deac077_Supplementary_Table_SIII [file deac077_supplementary_table_siii.pdf]

Human Reproduction, pp. 1–1, 2022
https://doi.org/10.1093/humrep/deac077

human reproduction

SUPPLEMENTARY DATA

Supplementary Table SIII A summary of unanswered questions regarding PCOS natural history from reproductive, psychological and oncological feature perspectives.

**General question (s)**	
**1**	What reproductive, psychological and oncological feature changes occur over time, in women with PCOS compared to those without PCOS across the life course?
**Specific question (s)**	
**1**	What are the changes in reproductive hormones (LH, FSH and LH/FSH ratio) and AMH over time in women with PCOS?
**2**	How does biochemical hyperangrodenism (such as total and free T, FAI and A_4) change over time across the life course?
**3**	Does mFG score change across the life course and across different ethnicity in women with PCOS and how would this impact diagnosis and later quality of life?
**4**	What are the changes in menstrual cycle regularity over time in women with PCOS?
**5**	Does the risk of endometrial or uterine cancer or breast cancer over time differ between women with and without PCOS?
**6**	Does the risk of depression and anxiety over time differ between women with and without PCOS?
**Other question (s)**	
**1**	What standard assays and cutoffs can be universally used in assessing biochemical hyperandrogenism?
**2**	What are the phenotypic variations in AMH levels over time in women with PCOS?
**3**	What should be the standard follow-up time for assessing each of the PCOS core outcomes?
